# Down syndrome and DYRK1A overexpression: relationships and future therapeutic directions

**DOI:** 10.3389/fnmol.2024.1391564

**Published:** 2024-07-24

**Authors:** Aidan J. Murphy, Steve D. Wilton, May T. Aung-Htut, Craig S. McIntosh

**Affiliations:** ^1^Centre for Molecular Medicine and Innovative Therapeutics, Murdoch University, Perth, WA, Australia; ^2^Perron Institute for Neurological and Translational Science, Centre for Neuromuscular and Neurological Disorders, The University of Western Australia, Perth, WA, Australia

**Keywords:** down syndrome, DYRK1A, antisense oligonucleotide, intellectual disability, exon skipping, DSCR

## Abstract

Down syndrome is a genetic-based disorder that results from the triplication of chromosome 21, leading to an overexpression of many triplicated genes, including the gene encoding Dual-Specificity Tyrosine Phosphorylation-Regulated Kinase 1A (DYRK1A). This protein has been observed to regulate numerous cellular processes, including cell proliferation, cell functioning, differentiation, and apoptosis. Consequently, an overexpression of *DYRK1A* has been reported to result in cognitive impairment, a key phenotype of individuals with Down syndrome. Therefore, downregulating DYRK1A has been explored as a potential therapeutic strategy for Down syndrome, with promising results observed from *in vivo* mouse models and human clinical trials that administered epigallocatechin gallate. Current DYRK1A inhibitors target the protein function directly, which tends to exhibit low specificity and selectivity, making them unfeasible for clinical or research purposes. On the other hand, antisense oligonucleotides (ASOs) offer a more selective therapeutic strategy to downregulate *DYRK1A* expression at the gene transcript level. Advances in ASO research have led to the discovery of numerous chemical modifications that increase ASO potency, specificity, and stability. Recently, several ASOs have been approved by the U.S. Food and Drug Administration to address neuromuscular and neurological conditions, laying the foundation for future ASO therapeutics. The limitations of ASOs, including their high production cost and difficulty delivering to target tissues can be overcome by further advances in ASO design. *DYRK1A* targeted ASOs could be a viable therapeutic approach to improve the quality of life for individuals with Down syndrome and their families.

## 1 Introduction

Down syndrome (DS) is the most common human chromosomal disorder, accounting for approximately 1 in 500 live births globally (Al-Biltagi, 2015). This disorder is caused by a partial or complete triplication of chromosome 21 and was first recognised by John Langdon Down (1887). Individuals with DS often present with characteristic facial features, intellectual disability (ID), hypotonia, muscle weakness, early-onset Alzheimer’s disease, increased incidence of leukemia, and heart deficits (Malt et al., 2013). While the presence and severity of these phenotypes may vary between individuals, almost all exhibit some degree of ID. The underlying cause of this is primarily the result of neurodevelopmental complications in specific brain regions. Unfortunately, there is currently no cure nor effective treatment for those with DS, leading many individuals to depend on familial or societal support for their lifetime. However, recent research has identified the *Dual specificity tyrosine phosphorylation regulated kinase 1 A* (*DYRK1A*) gene and protein as a potential therapeutic target for DS and other diseases due to its dose-dependent nature (Feki and Hibaoui, 2018; Liu et al., 2022). This gene, located at 21q22.13, plays a critical role in the development of the cognitive phenotypes associated with DS. Researchers have since been attempting to modulate the expression of the DYRK1A protein to recover one’s cognitive ability (Feki and Hibaoui, 2018). Currently available DYRK1A inhibitors such as epigallocatechin gallate (EGCG) derived from green tea or harmine, a hallucinogenic alkaloid, have shown a preliminary reduction in neurodevelopmental abnormalities across various animal models of DS and some human trials (Guedj et al., 2009; Adayev et al., 2011; Jarhad et al., 2018). However, these inhibitors are designed to non-specifically modify the protein’s function, increasing undesirable off-target effects, and rendering them unsuitable for DS intervention in humans.

In contrast, antisense oligonucleotides (ASOs) are emerging as a potential therapeutic modality for numerous genetic diseases that can be addressed by modulating gene expression. The specificity of the ASOs rely on Watson and Crick base-pairing to the target sequence and the oligomer chemistry then determines the subsequent mode of action. RNA or DNA-like oligomers, when annealed to a target RNA strand can induce degradation of the target through RNA-induced silencing complex or activation of RNase-H (Deleavey and Damha, 2012). In contrast, other oligomers with fully-modified bases and/or backbones can act as steric blockers and redirect normal protein translation, pre-mRNA splicing and polyadenylation (Iversen, 2001; Amantana et al., 2007; Liang et al., 2015; Wang et al., 2018; Scoles et al., 2019). Therefore, by altering the expression of *DYRK1A* at the level of RNA, we propose that ASOs could provide a highly specific and effective treatment option addressing ID and cognitive issues for those with DS, improving their overall quality of life.

This review will outline the current knowledge of DS, with a focus on its cognitive phenotypes and include a brief discussion on the debate surrounding the DS critical region (DSCR). This will be followed by a summary of *DYRK1A*, its role in DS and the DYRK1A inhibitors currently under investigation. Additionally, we will provide a brief analysis of ASOs with a focus on their mechanism of action and current U.S. Food and Drug Administration (FDA) approvals. The review will then conclude with an outline of some of the current challenges and strengths of developing and distributing ASOs. At present, there is a crucial need for novel therapies that can treat these individuals with high efficacy and low side effects. This review introduces the rationale behind the research intended to treat DS using ASO-based therapeutics.

## 2 Down syndrome

**Figure 1:**
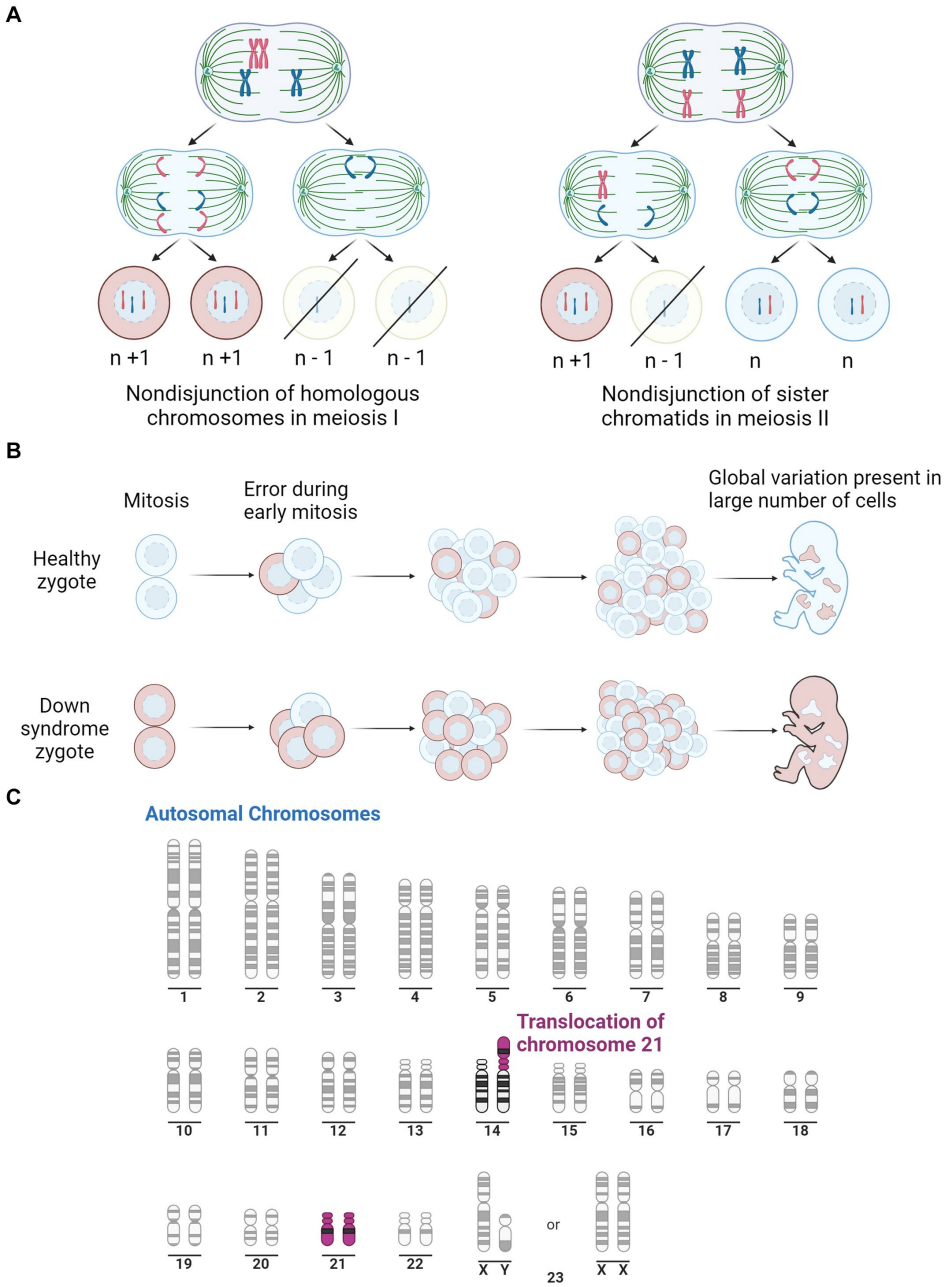
Illustration of the three pathways for Down syndrome development. **(A)** Representation of mitotic nondisjunction with the left being the most common form, resulting in a complete trisomy of chromosome 21. Where ‘*n*’ represents the typical number of chromosomes. **(B)** Is a representation of the partial trisomy of chromosome 21 through mosaicism which occurs from an early error in mitosis which results in a variation in chromosome number across the cells. Peach colour representing cells with typical number of chromosomes, light blue representing chromosomes with trisomy 21. **(C)** Is a representation of a second partial trisomy of chromosome 21 where chromosome 21 binds to chromosome 14, the most common site, leading to a partial trisomy in some cells. Purple represents Chromosome 21. Adapted from “Human Karyotype,” by BioRender.com (2023). Retrieved from https://app.biorender.com/biorender-templates.

The complete trisomy of chromosome 21 is the most common cause of DS and occurs from an error in cell division during the early development of the egg or sperm. It has been observed that approximately 88% of cases are of maternal origin, with incidences increasing with higher maternal age (Gómez et al., 2000; Fitzpatrick et al., 2017). Complete trisomy most often results from meiotic nondisjunction, accounting for 90–95% of DS cases (Papavassiliou et al., 2015). This occurs when the chromosomes fail to segregate to the opposite poles during meiosis, resulting in either trisomy or monosomy (Coppedè, 2016). Mosaicism can also result in DS, which accounts for 2–4% of DS cases, and causes a partial triplication of chromosome 21. Mosaicism can occur in two ways; after fertilisation, when an early mitotic error in an embryo results in the partial triplication of chromosome 21 (Papavassiliou et al., 2015); or it can occur in a DS zygote where a mitotic error causes some cells to revert to a normal karyotype (Papavassiliou et al., 2015). The remaining 2–4% of DS cases are caused by the inheritance of a chromosomal rearrangement/translocation which results in a partial triplication of chromosome 21. This occurs when chromosome 21 attaches to another chromosome, typically binding to chromosome 14 but can also bind to 13, 15, 21 or 22 (Bornstein et al., 2010). The various mechanisms for the development of DS are depicted in Figure 1.

### 2.1 Phenotypes

Those affected by DS present with a host of characteristic phenotypes and health complications that impede regular functioning, outlined in Table 1. These individuals have an increased risk of heart disease, early onset Alzheimer’s disease, leukemia and testicular cancer (Hill et al., 2003; Hasle et al., 2016). This not only disturbs the individual’s quality of life, but also puts a strain on family, carers and the health care system (Hill et al., 2003). While individual phenotypes vary, certain characteristics such as ID, hypotonia, craniofacial dysmorphology and the histopathology of Alzheimer’s disease, are present to some degree in all cases of DS. Understanding the key cognitive components of DS is crucial in comprehending the unique challenges of developing effective interventions that can improve their overall wellbeing.

**Table 1:** Outline of the common features observed in Down syndrome and incidence where applicable.

Features		Incidence (%)	References
Physical symptoms			
	Hypotonia (at birth)	70–76	Bull and Genetics (2011), Muthumani (2020)
	Flattened facial profile	51–89	Kava et al. (2004), Muthumani (2020)
	Malformed head	45	Muthumani (2020)
	Ear abnormalities	67	Kava et al. (2004)
	Upward slanting eyes	84–86	Kava et al. (2004), Muthumani (2020)
	Excess skin at nape of neck	36.8	Kava et al. (2004)
	Simian crease (unilateral or bilateral)	33.2	Bull and Genetics (2011)
	Bushfield spots on iris	3.2	Kava et al. (2004)
Cognitive symptoms			
	Attention problems		Silverman (2007)
	Poor working memory capacity		Silverman (2007)
	Memory impairment		Silverman (2007)
	Highly sociable		Fidler et al. (2008)
	Learning deficit		Silverman (2007)
	Delayed language and speech development		Silverman (2007)
Associated medical conditions			
	Heart disease	50	Bull and Genetics (2011)
	Dementia (by 70 years old)	90	Ballard et al. (2016)
	Serious otitis media	50–70	Bull and Genetics (2011)
	Ocular deficits	60	Bull and Genetics (2011)
	Gastrointestinal defects	12	Bull and Genetics (2011)
	Hearing loss	75	Bull and Genetics (2011)
	Abnormal immune response		Ram and Chinen (2011)
	Epilepsy	8–26	Lefter et al. (2011), Vignoli et al. (2011)
	Hyperthyroidism	15	Bull and Genetics (2011)
	Leukemia	2–2.5	Baruchel et al. (2023)

### 2.2 Cognition

Individuals with DS account for approximately 10–20% of the intellectually disabled population, with some form of impairment present in nearly all cases of DS (Torr et al., 2010). This is the result of overexpressed genes altering neural development and functioning. Individuals with DS generally have an intelligence quotient (IQ) that ranges from 20 to 80. Their IQ being its highest when they are children and gradually declines as the individual ages (Carr, 1988; Anneren and Edman, 1993; Chapman and Hesketh, 2000). This is believed to be a consequence of the slow rate of neural development when compared to unaffected children (Carr, 1988; Chapman and Hesketh, 2000). By adulthood, an individual with DS is likely to have an IQ of 25 to 55, equating their mental age to be approximately 7–8 years old (Pennington et al., 2003).

The specific cognitive shortfalls associated with DS include deficits in memory, language, speech, hearing, processing speed and numerous aspects of executive function (Silverman, 2007; Lanfranchi et al., 2010). In early childhood, ID is not as prominent, with steady cognitive decline being observed from late childhood into early adulthood, with a further decline in middle to late adulthood, typically associated with early onset Alzheimer’s disease (Grieco et al., 2015). Interestingly, there is a discrepancy between cognitive tasks, with verbal-based tasks being performed worse than non-verbal-based tasks (Abbeduto et al., 2001; Couzens et al., 2011; Channell et al., 2014). Language skills until age 5 are generally within the expected developmental range, however, steadily decline into adulthood (Guralnick, 2002). These language shortfalls have been hypothesised to be a secondary consequence of the deficit in verbal working memory more specifically, in the phonological loop located in the temporal lobe (Chapman, 2006; Vicari and Carlesimo, 2006). Additionally, those with DS have been shown to exhibit numerous deficits in executive function, including impairments of attention, inhibition, processing speed, multi-tasking, self-monitoring, working memory and organisation (Grieco et al., 2015). These executive function skills are processed in the frontal lobes and have a secondary impact on one’s learning ability. Furthermore, learning and memory deficits are believed to result from errors in the encoding and retrieval of memories, which are processed in the temporal lobe and the hippocampus (Carlesimo et al., 1997). While there is some debate in the literature over the underlying mechanisms that result in these cognitive deficits, there is largely an agreement that the individual deficits in each cognitive area are largely of primary origin, with some top-down cascade also influencing cognitive functioning (Grieco et al., 2015). This conclusion has been supported by studies that identify alterations in brain structure in similar brain regions.

### 2.3 Alterations in brain structure

Deficits in cognition are almost always the result of abnormal brain structure, with global cortical volume loss often being correlated with cognitive decline (Jack et al., 2008; Soria-Pastor et al., 2008; Calabrese et al., 2011; Mcdonald et al., 2012). Consistently, the brains of individuals with DS are smaller when compared to healthy individuals, with a study on children finding a 13.3% reduction in total brain volume (Rachidi and Lopes, 2011; Śmigielska-Kuzia et al., 2011). This has been observed to increase to 20% in adults with DS (Kemper, 1991). Interestingly, this global reduction in brain volume has not been observed in trisomic mouse models (Contestabile et al., 2010). Some suggest that these findings indicate that volume is not directly related to cognitive impairment. However, we postulate that this may be a result of the difference in neuronal complexities between mice and humans, resulting in more severe and widespread disability in humans due to the complexity of our neuronal development when compared to that of mice. Global cortical volume loss often results from a catastrophic cascade of errors that leads to drastically reduced neuronal cell numbers. With that said, early DS mouse models exhibited increased ventricle size and brachycephaly (Guedj et al., 2012). These phenotypes typically indicate some form of cortical atrophy, which may be attributed to improper neurodevelopment or neuronal cell death that has also been observed (Arbones et al., 2019).

**Figure 2:**
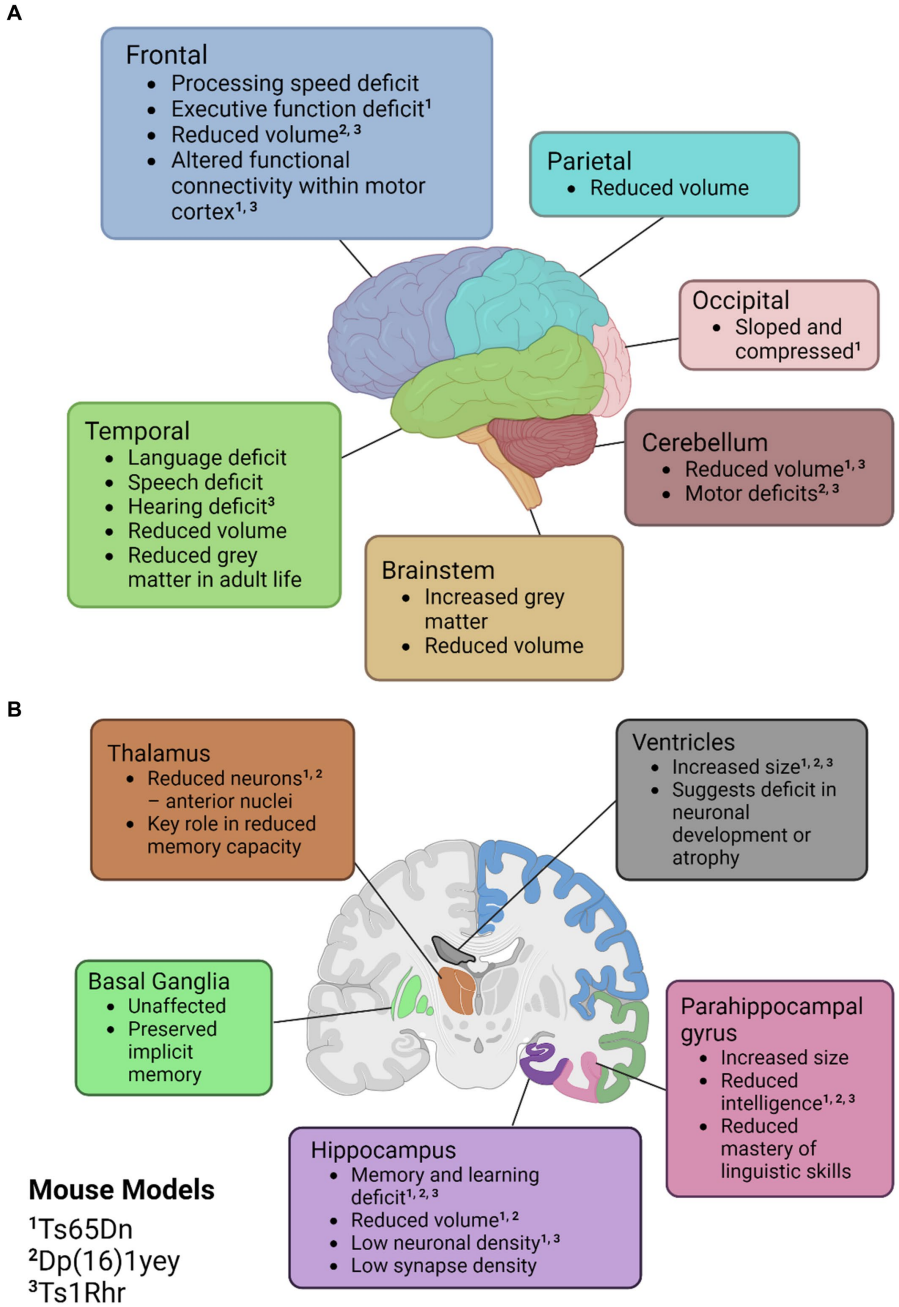
Illustrations of the neuroanatomical and cognitive features of Down syndrome. **(A)** Cortical mapping of affected regions. **(B)** Coronal section revealing the subcortical structures influenced by Down syndrome. Mouse models with known similar phenotypes have been identified with superscripts; ‘1’ for Ts65Dn, ‘2’ for Dp(16)1yey, and ‘3’ Ts1Rhr. Created with BioRender.com.

Across the literature, the primary regions affected in mice are the cerebellum and the hippocampus. Some mouse models have detected a cerebellar volume loss of up to 12% and in models that do not exhibit a volume loss, a lower density of neuronal cells is still observed (Baxter et al., 2000; Olson et al., 2004a). A well-characterised mouse model for DS is the Ts65Dn mouse which is trisomic for approximately two-thirds of the genes orthologous to human chromosome 21 (Hsa21). Interestingly, in Ts65Dn mice, there is no alteration in hippocampal volume at 7 months of age and no alteration in neuronal density at one month of age (Lorenzi and Reeves, 2006; Olson et al., 2007). However, at 16–17 months old, neuronal density has been found to be significantly lower in the cornu ammonis (CA) 1 region of the hippocampus and synapse density has also been found to be reduced in the dentate gyrus, CA1 and CA3 when compared to diploid mice (Kurt et al., 2004; Lorenzi and Reeves, 2006). This is similar to what is observed in humans, where these regions have been reported to have a reduced volume of the brainstem, frontal, temporal and parietal lobes. It is suggested that this is indicative of a decreased formation of new neurons during development and increased atrophy in adult life. An overview of the common neuronal features of DS in humans can be found in Figure 2. Additionally, in this figure we have indicated similar phenotypes observed in the murine models previously mentioned.

Studies on humans with DS have noted similar abnormalities to the murine models listed in comparable brain regions. Evidence of improper cortical development from DS-affected brains have been observed at the cellular level with abnormal dendrite formation, impaired neurogenesis, lower neurotransmitter counts, and reduced synaptic proteins (Vacca et al., 2019). Notably, dendritic abnormalities in the hippocampus and the dentate gyrus appear to be the most pervasive, with murine models exhibiting reduced synaptic density, altered dendritic arbours, and altered dendritic spines (Contestabile et al., 2010). Numerous other alterations in neurotransmitter systems, cellular mechanisms, and degradation due to age-associated dementia are outlined in the literature. However, these are outside of the scope of this review, but have been reviewed by Arbones et al. (2019) and Vacca et al. (2019). The previously mentioned alterations in brain morphology are common hallmarks of many forms of ID and provide the morphological basis for the poor cognition observed in DS individuals. Consistent neurobiological abnormalities had sparked interest in the possibility of a critical region of chromosome 21 that may be present in all cases of DS, providing a rationale for the main phenotypes and potential therapeutic target.

### 2.4 Down syndrome critical region

**Figure 3:**
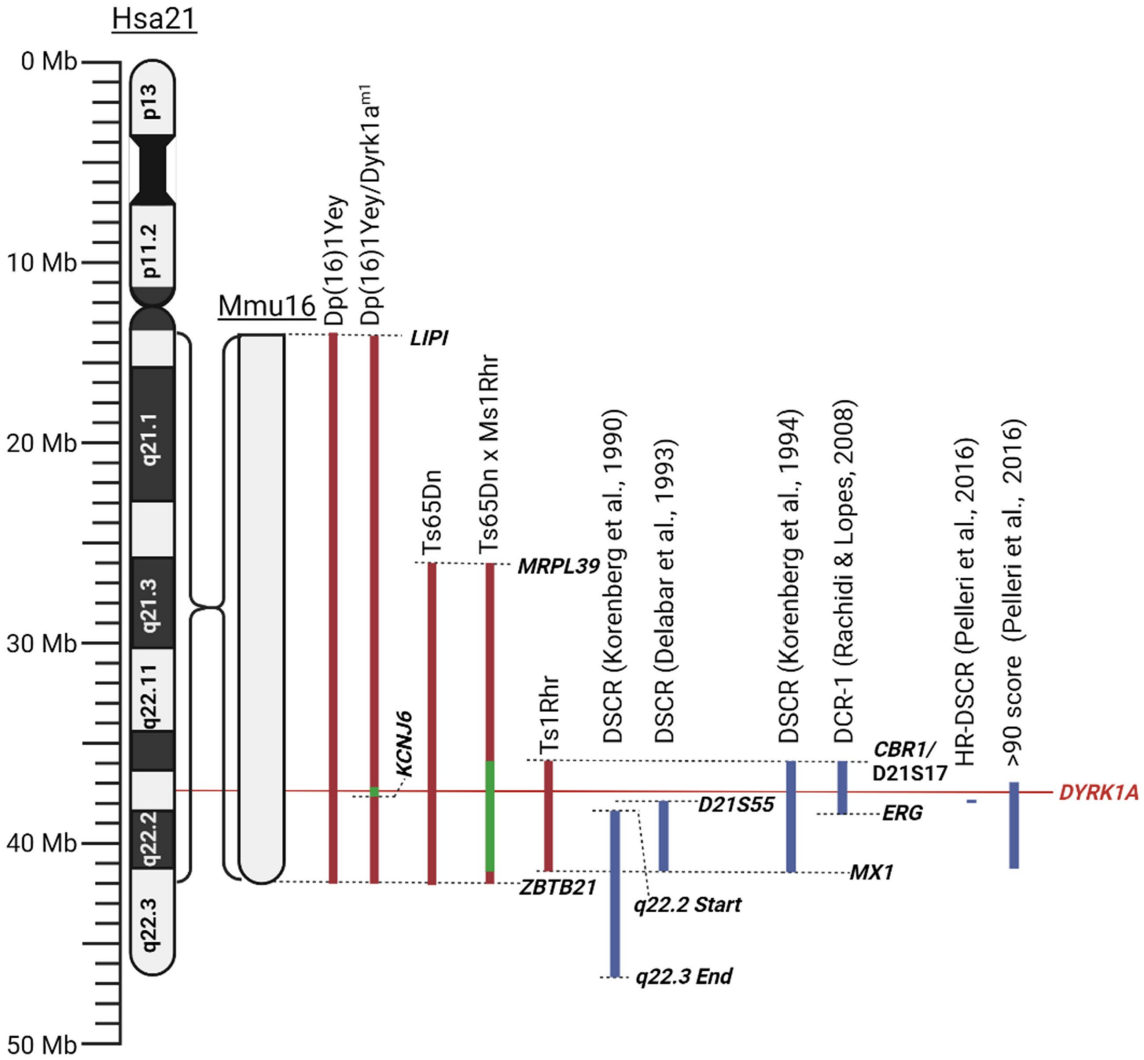
Schematic of mouse mutants aneuploid segments and Down syndrome critical regions described in this literature review. Human chromosome 21 (Hsa21) and the orthologous region of mouse chromosome 16 (Mmu16) are shown. Dashed lines identify locations of specific genes or chromosomal landmarks. All mouse models are aligned according to Hsa21, and the Mmu16 chromosome segment is scaled according to Hsa21. Red indicates trisomic sections, green indicates haploid sections, and blue indicates hypothesised Down syndrome critical regions. Created with BioRender.com.

As there exists both a complete and partial trisomy of chromosome 21 in DS populations, the concept of a DSCR has been debated amongst the literature. Korenberg et al. (1990) hypothesised a DSCR that extended from the start of q21.2 to the end of chromosome 21. This was heavily based on earlier literature that pinpointed a crucial area on the distal end of chromosome 21. This was later refined by Delabar et al. (1993) using genotype–phenotype analysis of 10 people with partial trisomy 21 to the region D21S55 (~37.8 Mb) to MX1 (~41.7 Mb). They hypothesised that this region is responsible for 19 of the 33 phenotypes they assessed. Another paper by Korenberg et al. (1994) emphasised the importance of genes outside the D21S55 region and, through their own molecular and phenotypic analysis, defined a region with a proximal boundary of D21S17 (~35.9 Mb) and a distal boundary at MX1. All DCSRs mentioned can be seen in Figure 3 (Korenberg et al., 1990; Delabar et al., 1993; Korenberg et al., 1994; Yamamoto et al., 2011; Schnabel et al., 2018).

Since its conception, the idea of a DSCR has been heavily debated, with some reviews and more recent literature finding their own definitions of the region, such as in the review by Rachidi and Lopes (2008). This led to the question of whether it is valid to conclude that such a region exists at all. Recent research has failed to support the prevalence of a DSCR, as per the original Korenberg et al. (1990) hypothesis. Two critical studies on mice had shown that the triplication of the DSCR alone does not result in the characteristic facial or neurological DS phenotypes (Olson et al., 2004b, 2007). The physical phenotypes observed when comparing DS mice (Ts65Dn) and triplicated DSCR-only mice (Ts1Rhr) presented an opposite phenotypic pattern, where Ts65Dn mice were smaller while Ts1Rhr mice were larger (Olson et al., 2004b). Additionally, neurological characteristics like cerebellar volume, hippocampal size and function were either not as severe or protected in Ts1Rhr mice, suggesting that the DSCR proposed in the original hypothesis does not fully recapitulate the main DS phenotypes (Olson et al., 2007). However, some caution is advised when drawing conclusions based on this study as the orthologous region of Hsa21 in murine models is located across three separate chromosomes, potentially adding some variability. Additionally, animal models may require multiple copies to replicate the human phenotype, such as in experiments involving SOD1 replicating motor neuron disease (Ferraiuolo et al., 2007; Nardo et al., 2016). Future studies should be conducted with more copies of the DSCR to see if phenotypes differ. Additionally, the researchers bred trisomic Ts65Dn mice with monosomic DSCR Ms1Rhr mice, producing offspring that were trisomic for chromosome 21 with a normalised analogous DSCR (Olson et al., 2007). These DSCR-normalised mice were found to perform similarly in the Morris water maze when compared to a control euploid mouse (Olson et al., 2007). These researchers agree that the DSCR hypothesis has been disproved, however, they concluded that the genes within the DSCR are necessary but not sufficient to produce a learning deficit in DS (Olson et al., 2007). Most importantly, this suggested that a return to normal gene expression in DSCR genes may induce an improvement in cognitive functioning. Contradicting these studies, other researchers concluded that the DSCR is sufficient to produce the DS phenotype, coming to this conclusion as Ts1Rhr mice were found to significantly differ on 20 of 48 characteristics when compared to control ‘2 N’ mice (Belichenko et al., 2009). These included altered long-term potentiation effects, dendritic spine enlargement and density in the fascia dentata, among others (Belichenko et al., 2009). While they concluded that the triplicated region present in Ts1Rhr mice is sufficient to reproduce the DS phenotype, they also mention that differing combinations of single or multiple gene dosage effects may give rise to different phenotypes. This is very similar to a more recent ‘gene dosage effect’ hypothesis proposed by Moreau et al. (2021). This hypothesis suggests that DS results from an imbalance in gene dosage, leading to the overexpression of specific causative genes, which can alter interactions between other genes in the genome. Unlike the DSCR hypothesis, which attributes the DS phenotypes solely to the genes within a specific region. The gene-dosage effect hypothesis considers a broader range of genetic interactions across the entire genome that are responsible for the DS phenotypes. We consider this to be more plausible than the original DSCR hypothesis. However, the gene-dosage hypothesis requires more sophisticated methods to identify the most relevant genes involved in DS.

Pelleri et al. (2016) conducted a thorough review which detailed the score for association with DS of numerous individuals with partial trisomy 21 to identify a highly restricted DSCR (HR-DSCR). This region is defined by a triplication of genes present in all DS cases and absent in all non-DS cases. They identified the HR-DSCR region as genes with a prevalence score over 97. However, it contained genes only homologous to the chimpanzee genome that have not been thoroughly researched. We suggest that it would be more useful to direct future exploration toward genes just outside this HR-DSCR that are homologous to more common disease models. The small region with a score over 90 includes nearly the entire q22.13 segment (Pelleri et al., 2016). A high score in this study indicates an increased probability of association with DS. This region includes seven protein coding genes: *DYRK1A, DSCR3, TTC3, PIGP, RIPPLY3, KCNJ15, KCNJ6*, and *DSCR4*. Of these the former 5 are expressed in the adult brain and are potential targets for DS treatment. Given the wealth of literature and its potential implications for various diseases, our review will focus on the gene *DYRK1A*. From the Pelleri et al. (2016) study, this gene has a prevalence score of 91 out of 100 for its association with DS. Additionally, DYRK1A has been consistently overexpressed in DS human and mouse models and has been found to play a vital role in neural function, processing and development (Guimera et al., 1999; Dowjat et al., 2007). Interestingly, a recovery in the developmental cognitive deficit was reported after a partial rescue of *DYRK1A* in DS mice. Researchers utilised mice with a gene trap vector inserted in intron 4 resulting in disruption of the 321 amino acid kinase domain resulting in a haploinsufficiency of DYRK1A and was referred to as Dyrk1a^m1^ (Jiang et al., 2015). They bred this mouse with their DS mouse model, Dp (16)1, to generate a DS mouse with a normalised *DYRK1A* expression, Dp (16)1/Dyrk1a^m1^ (Jiang et al., 2015). These mice exhibited performance improvements in T-maze and contextual fear-conditioning tests when compared to Dp (16)1 mice (Jiang et al., 2015). Thereby supporting the potential causative role of *DYRK1A* in the cognitive phenotype and potential for recovery if the *DYRK1A* gene is normalised. All the chromosomal segments for the murine models are presented in Figure 3.

The conclusion of much of the literature appears to be that a strictly defined DSCR does not exist, and while there are certain genes that appear necessary to produce the phenotypes of DS, these are not sufficient when viewed in isolation. Therefore, we also agree that the restrictive DSCR approach is not adequate to explain the phenotypic outcomes of DS. Importantly, the recovery of the widely researched gene *DYRK1A* has shown to alleviate the severity of cognitive phenotypes in DS models, making the regulation of these genes a promising potential therapeutic strategy.

## 3 DYRK1A

**Figure 4:**
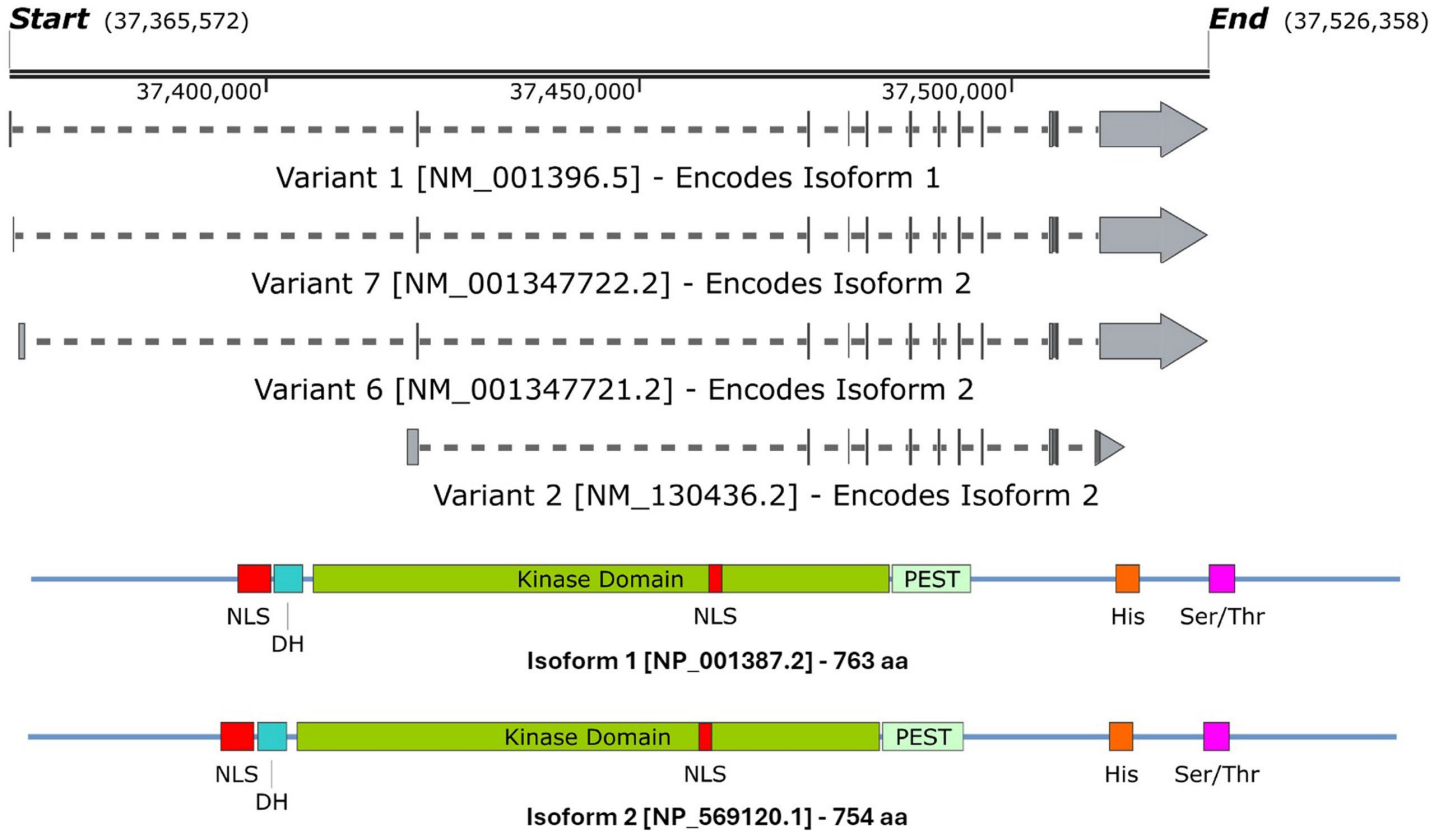
A schematic representation of **(A)** the *DYRK1A* mRNA transcripts that encode **(B)** isoforms 1 & 2 DYRK1A proteins. Both isoforms share the same features and domains but differ in amino acid coordinates. The amino acid positions of each domain/feature are specified for isoform 1. These include: two nuclear localisation signals (p117–135 & p380–386, NLS); a DYRK homology box (p137–152, DH); a PEST-rich region (p482–525, PEST); a polyhistidine stretch (p607–619, His); and a serine and threonine rich region (p659–672, Ser/Thr). Created with SnapGene software (www.snapgene.com).

*DYRK1A* has been found to play a vital role in the regulation and functioning of the processes involved in neurodevelopment (Olson et al., 2004b, 2007). The first evidence was studied in *Drosophila* mini brain (mnb) mutants which exhibited altered neural proliferation and smaller brain size (Tejedor et al., 1995). *Mini brain* (discovered in insects) is an orthologous gene to the vertebrate *DYRK1A*; hence forth in this review, *mnb*/*DYRK1A* will only be referred to as *DYRK1A* (Tejedor and Hämmerle, 2011). Among others in the DYRK family, *DYRK1A* is activated via tyrosine autophosphorylation in the activation loop but phosphorylates its substrates on serine and threonine residues only (Lochhead et al., 2005). However, DYRK1A is unique in that it is the most ubiquitously expressed when compared to other DYRK members. These other DYRK’s are often more restricted and often highly expressed in the testes and muscle (Becker et al., 1998; Leder et al., 1999; Zhang et al., 2005; Sacher et al., 2007). Multiple *DYRK1A* transcripts exist through alternative splicing and untranslated region changes Figure 4A, which in turn encodes two main DYRK1A protein isoforms Figure 4B. It should be noted that there are other shorter isoforms that are reported via internal splicing events, however, there is currently no evidence of a functional difference between these. Examining the protein isoforms 1 and 2, DYRK1A exhibits a conserved N-terminal motif that stabilises the kinase domain during protein maturation (Soundararajan et al., 2013). This N-terminal is shared amongst the other members of the DYRK family and is commonly referred to as the DYRK homology box (DH). DYRK1A exhibits a two nuclear localisation signals (NLS) one next to the DH and the other within the kinase domain. The main DYRK1A isoforms also exhibit PEST, polyhistidine (His) and serine-threonine rich (Ser/Thr) motifs.

**Figure 5:**
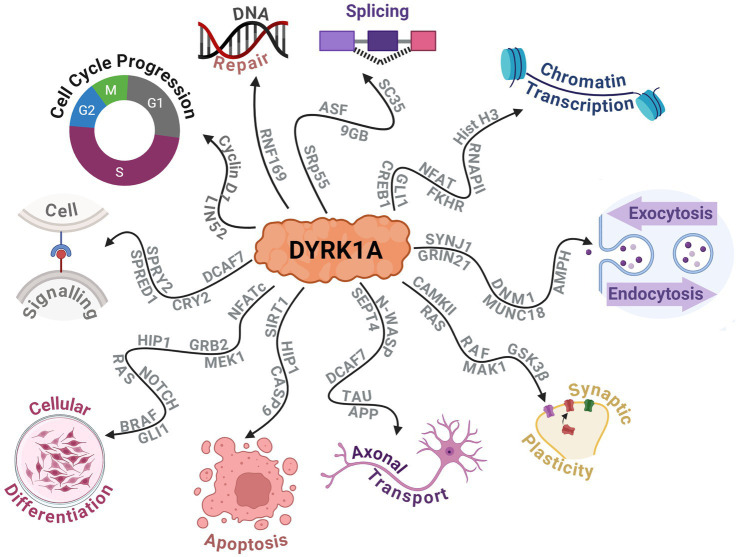
Schematic displaying the protein–protein interactions of DYRK1A and the widespread downstream molecular functions that are affected. Created with BioRender.com.

DYRK1A has been found to exhibit numerous effects on key aspects of the central nervous system (CNS) such as synaptic plasticity and neuronal differentiation (Arbones et al., 2019; Atas-Ozcan et al., 2021; Ananthapadmanabhan et al., 2023). Additionally, it plays a broader role in cellular development, function and repair. This includes vital functions in cell cycle progression, splicing, chromatin transcription, cell signalling, exocytosis, endocytosis and apoptosis. Recent research highlights DYRK1A’s role in modifying RNF169, which is crucial for DNA repair following damage (Guard et al., 2019). The protein interactions that DYRK1A alters to produce these effects are outlined in Figure 5. Additionally, the effect on neurodevelopment was further observed by a study on haploinsufficient *DYRK1A*^+/−^ mice that had smaller brains and fewer neurons when compared to wild-type littermates (Fotaki et al., 2002). Additional studies have noted that altered *DYRK1A* expression influenced neural numbers, neurogenesis, synaptogenesis, neural functions, and neurotransmission across various human and murine models (Arbones et al., 2019). Expression of *DYRK1A* across the lifespan in mice is relegated primarily to the CNS. It has been found to peak near birth during neuronal dendritic morphogenesis and later maintained at lower levels in adulthood (Okui et al., 1999). This provided evidence of its crucial role in neurodevelopment and maintenance and has shown that an alteration in *DYRK1A* expression levels can greatly affect the individual’s neural functioning.

### 3.1 Dose sensitivity

In humans, chromosome 21 contains over 300 genes, with only one-third found to be dose-sensitive and are hypothesised to be the primary genes associated with the DS phenotype (Yahya-Graison et al., 2007; Becker et al., 2014). *DYRK1A* is one of these dose-sensitive protein-coding genes that, if over or under-expressed, can affect essential cellular development and functional roles depending on the pattern of expression. Duchon and Herault (2016) postulated that the formation of active DYRK1A protein complexes may be the cause of the dose sensitivity (Duchon and Herault, 2016). Whereby the dosage sensitive protein forms a tripartite complex with two partners (Veitia et al., 2008; Duchon and Herault, 2016). This hypothesis was corroborated by co-immunoprecipitation studies that identified DYRK1A complexes that formed with cytoskeleton filamentous actin, neurofilaments and tubulin (Kaczmarski et al., 2014). However, the symptoms observed when DYRK1A is under-expressed differ from those when it is over-expressed, contrary to what this tripartite model may suggest (Arque et al., 2013; Raveau et al., 2018). Additionally, numerous DS studies and transgenic models overexpressing DYRK1A have found that DYRK1A protein activity is increased, contradicting this model. These findings suggest that even if a tripartite system exists, it likely does not act alone (Ryoo et al., 2007; Wegiel et al., 2011; Kim et al., 2016). Therefore, the DYRK1A system is likely to be more complex, with other mechanisms influencing symptomology. Further research may elucidate this underlying mechanism, as this is speculation at the time of writing.

#### 3.1.1 Under-expression in DYRK1A syndrome

Heterozygous disruption or mutations causing a loss of function can result in a rare partial monosomy of chromosome 21 known as *DYRK1A* Related Intellectual Disability Syndrome (DYRK1A Syndrome), also referred to as Autosomal Dominant Mental Retardation 7. These individuals often present with numerous developmental delays, ID, dysmorphic facial features, autism spectrum disorder, microcephaly, and a high frequency of epileptic seizures (Courcet et al., 2012; Bronicki et al., 2015; Ruaud et al., 2015; Van Bon et al., 2016). A critical study by Matsumoto et al. (1997) narrowed the loci of the microcephaly and intrauterine growth retardation to a segment of 21.q22.2 that’s 1.2 Mb long that includes *DYRK1A* and several other genes. Further exploration into DYRK1A syndrome is outside the scope of this review. For more information, we recommend the review by van Bon et al. (2021).

#### 3.1.2 Over-expression in down syndrome

The triplication of chromosome 21 in humans results in an over-expression of the genetic information retained within it. Lymphoblastoid cells retrieved from humans with DS exhibited an approximate 1.4-fold increase in *DYRK1A* expression (Yahya-Graison et al., 2007). Similarly, studies on Ts65Dn mice analysed their DYRK1A protein expression in the cortex, hippocampus and cerebellum and observed an approximate 1.6-fold increase in expression across all regions (Souchet et al., 2014). This expression pattern has been echoed in mice triplicated for *DYRK1A* alone, BACTgDyrk1a. However, the relative expression was found to vary depending on the brain region, with a 1.6-fold increase in the cortex, a 1.9-fold increase in the hippocampus and a 1.7-fold increase in the cerebellum (Guedj et al., 2009, 2012).

Mouse models bred to exhibit three functional copies of *DYRK1A* have shown some neurodevelopmental deficits and cognitive phenotypes similar to DS models. For example, TgDyrk1a mice have displayed difficulties in locomotion, negative geotaxis, and spontaneous alternation (Arque et al., 2013). Numerous studies have also reported developmental and functional changes in the murine and human brains, including suppression of cortical neurogenesis (Chakrabarti et al., 2007), increased ventricles (Schimmel et al., 2006), increased inhibitory interneurons (Pérez-Cremades et al., 2010), and altered dendrites (Dierssen and Ramakers, 2006), long term potentiation and long term depression in prefrontal cortex (Souchet et al., 2014; Thomazeau et al., 2014). Interestingly, there has been found to be an inversely correlated relationship between *DYRK1A* expression and neuron numbers in the neocortex, while there still exhibits a positive correlation in other brain regions (Guedj et al., 2012). This highlights the region-specific nature of *DYRK1A* and its importance in neuronal development.

Furthermore, Ts65Dn DS mice crossbred with heterozygous *DYRK1A*^+/−^ mice produced a DS mouse model with a normalised *DYRK1A* expression level. The results of studies using these mice found that the long-term potentiation in the hippocampus was protected, early neurogenesis was increased, and Cyclin D1 was recovered (García-Cerro et al., 2014; Najas et al., 2015), providing evidence that *DYRK1A* is necessary in the development of these phenotypes. Importantly, pharmacological inhibition of *DYRK1A* has shown to exhibit similar deficit recovery, suggesting that normalising the gene’s expression could correct adverse phenotypes.

### 3.2 Current DYRK1A inhibitors

The dose-dependent nature of *DYRK1A* has made it an attractive target gene and protein for therapeutic intervention, resulting in the development and discovery of numerous pharmacological therapies. Given the extensive range of DYRK1A inhibitors currently available, we will briefly outline the most notable ones, with a primary focus on those aimed at alleviating neurological deficits.

#### 3.2.1 Epigallocatechin gallate

The first DYRK1A inhibitor in animals and humans was identified as EGCG, which is derived from green tea (Jarhad et al., 2018). It inhibits the DYRK1A protein with high potency (IC_50_ = 330 nM), albeit with low specificity—as it has also been found to inhibit p38-regulated/activated kinase among numerous others (Lamoral-Theys et al., 2010). This has significantly impacted the ability to establish firm conclusions regarding a causative influence on *DYRK1A* expression. However, it is a common supplement with a high safety profile that has still been used in studies on humans. Mice overexpressing *DYRK1A* were administered EGCG orally and exhibited improved structural development and cognitive abilities (Guedj et al., 2009). Additionally, it has been given to human young adults with DS and improved hippocampal functioning, particularly in visual and spatial working memory-based tasks (De La Torre et al., 2014). A phase II clinical study showed similar results, with the EGCG group performing better in some cognitive tests and adaptive behaviour up to 12 months post-treatment (De La Torre et al., 2016). Curiously, evidence suggests that EGCG does not cross the blood brain barrier (BBB) effectively, which makes these previous findings peculiar (Becker et al., 2014). It has been suggested that this may be due to the other beneficial effects of EGCG, such its antioxidant effects or its effects on other proteins (Becker et al., 2014). Additionally, recent discoveries have noted that relatively strong doses of EGCG administered to mice at early life could disrupt facial development and, in some cases, cause more severe facial dysmorphia (Starbuck et al., 2021). This could indicate that very low *DYRK1A* have caused these undesirable effects. However, a more likely hypothesis is that these result from the non-specific nature of EGCG.

#### 3.2.2 Harmine

Harmine is a β-carboline alkaloid initially isolated from a South American vine and was found to be a potent inhibitor of DYRK1A (IC_50_ = 80 nM). However, it also inhibited monoamine oxidase A (*MAO-A*), and other members of the DYRK family, particularly *DYRK2* (IC_50_ = 0.9 μM), and *DYRK3* (IC_50_ = 0.8 μM) (Bain et al., 2007). Like many other DYRK1A inhibitors, Harmine and its derivatives work via competing with ATP binding to DYRK1A, which inhibits serine/threonine phosphorylation activity (Adayev et al., 2011). Due to its potent inhibition of *MAO-A*, numerous derivatives have been designed to increase the selectivity to DYRK1A. However, not all have been successful and still exhibit inhibition of *MAO-A* to some degree (Jarhad et al., 2018).

#### 3.2.3 Other inhibitors

A non-exhaustive list of the various DYRK1A inhibitor categories has been included in Table 2. Jarhad et al. (2018) and Liu et al. (2022) provide comprehensive reviews on these and more DYRK1A inhibitors. Currently, the field of DYRK1A inhibitor research is in a state of development. The existing inhibitors have been observed to exhibit significant off-target effects, primarily due to DYRK1A being highly homologous with numerous other kinases, particularly of the CMGC family. The off-target effects render these inhibitors unviable for clinical use and may result in inconclusive findings in research. Additionally these inhibitors display numerous issues with drug metabolism, which can include rapid degradation, low metabolic stability or low BBB permeability (Liu et al., 2022).

**Table 2:** Outline of the various DYRK1A inhibitor types outlining the number of common variants and/or structural analogues from Jarhad et al. (2018).

DYRK1A Inhibitor Type		References
Naturally occurring		
	EGCG	Singh et al. (2011)
	Harmine & Derivatives	Zhang et al. (2020)
	Acrifoline	Beniddir et al. (2014)
	Leucettines	Naert et al. (2015)
	Meridianins	Yadav et al. (2015)
	Staurosporine	Alexeeva et al. (2015)
Synthetic		
	Benzothiazoles	Rothweiler et al. (2016)
	Indolocarbazole	Sánchez et al. (2009)
	Indazole	Hood et al. (2017)
	Benzimidazoles and Imidazopyridines	Hulme et al. (2020)
	Azaindoles	Gourdain et al. (2013)
	Purine Derivatives	Demange et al. (2013)
	Thiazoloquinazoline Derivatives	Foucourt et al. (2014)
	Naphthyridines	Grygier et al. (2023)
	β-Carboline Derivatives	Frost et al. (2011)
	Quinoline Derivatives	Falke et al. (2015)
	Quinazoline Derivatives	Tazarki et al. (2019)
	Pyrimidine Derivatives	Li et al. (2016)
	Pyridazine Derivatives	Bruel et al. (2014)
	Polyphenol derivatives	Araldi and Hwang (2022)

This is where ASOs may offer a revolutionary treatment option as they can be designed to specifically target the *DYRK1A* gene transcript and modulate expression of the protein. Additionally, ASOs designed to treat other CNS-based disorders have shown stability in the CNS with several having received FDA approval (Goodkey et al., 2018; Wilton-Clark and Yokota, 2021; Eser and Topaloğlu, 2022; Patterson et al., 2023; Van Roon-Mom et al., 2023). Should a DYRK1A inhibitor become available, this would not be limited to a treatment for DS as it would have benefits for cancers, Alzheimer’s disease, viral infections, heart disease, Huntington’s disease, among others (Deboever et al., 2022). Therefore, individuals and researchers would benefit immensely from the production of a highly selective and specific DYRK1A inhibitor.

## 4 Antisense oligonucleotides

Antisense oligonucleotides are short (~12–30 nucleotides long) synthetic nucleic acid analogues that can be used to alter gene expression via hybridisation to a complementary DNA or RNA through Watson-Crick base pairing (Crooke et al., 2021). First discovered by Zamecnik and Stephenson (1978), they noticed that ASOs inhibited viral replication *in vitro*. However, before the late 1980s, no effort was made toward a medicinal use for oligonucleotides. Since then, considerable effort has been made to improve upon every facet of ASO technology, aside from those required for Watson-Crick base pairing. This has amounted to many analogues being synthesised and evaluated. Critical strategies for enhancing these chemistries safety and efficiency mainly involved the modifications of the phosphodiester backbone and the 2′ position of the sugar moiety and eventually the creation of the neutrally charged, synthetic phosphorodiamidate morpholino oligomer (PMO). Several other mechanisms of manipulating gene expression include transcription blocking (Melton, 1985), polyadenylation blocking (Vickers et al., 2001), small interfering RNAs (Foster et al., 2018), translational blocking (Setten et al., 2019), and gene therapies (Anguela and High, 2019). However, exploration into these is outside the scope of this review.

### 4.1 Mechanism of action

The mechanistic action of an ASO is largely dependent on its chemistry and the region of mRNA in which it is designed to anneal, which can be split into two groups: occupancy-mediated degradation and occupancy-only mechanisms, also known as steric interference. Depending upon the base modifications, phosphorothioate (PS) ASOs can be designed to exploit both groups of mechanisms, while PMOs do not support occupancy-mediated degradation.

#### 4.1.1 Occupancy-mediated degradation (RNase-H)

**Figure 6:**
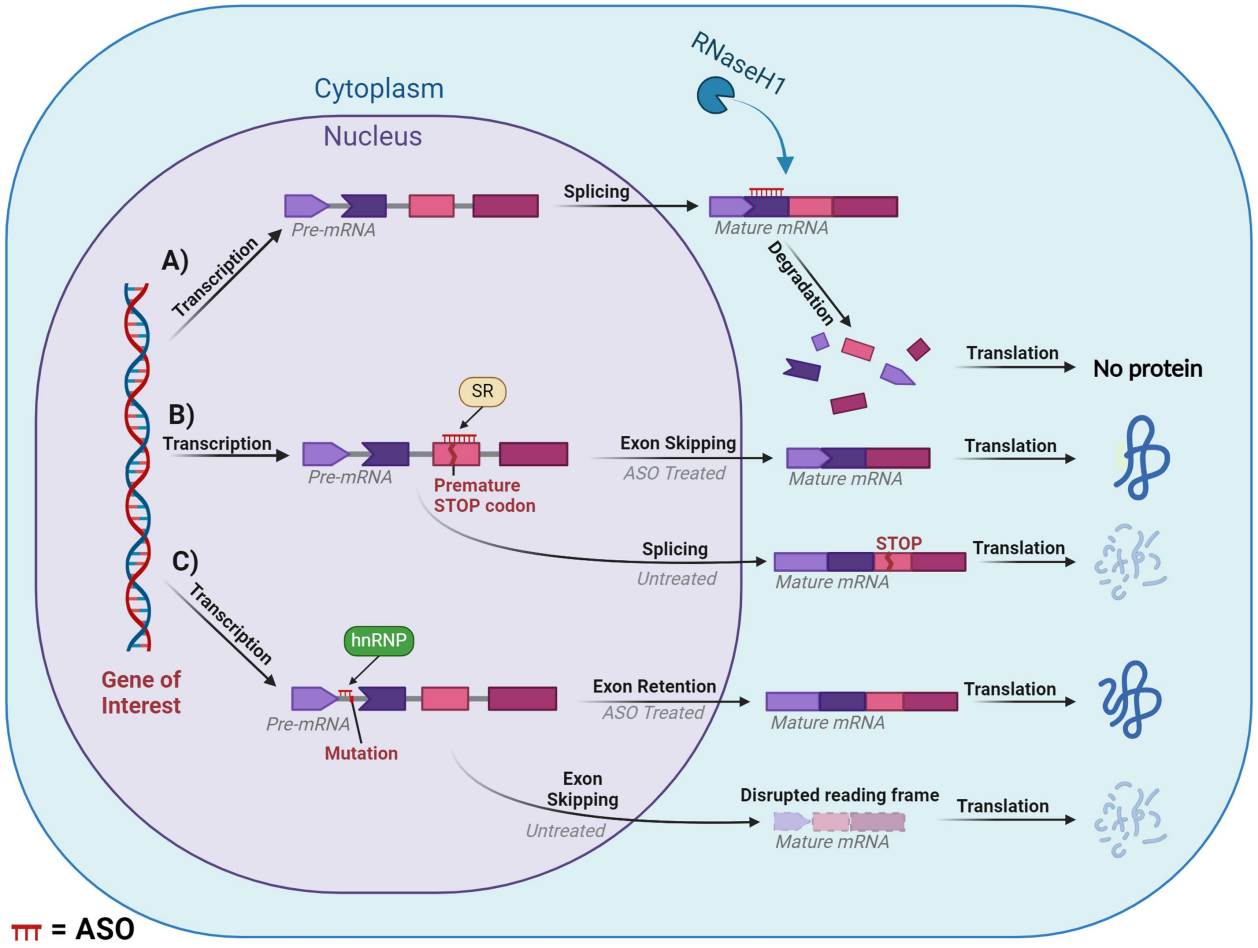
Diagram of splice modulation mechanism of action of antisense oligonucleotides (ASO). **(A)** RNase-H mediated degradation. **(B)** Splice-switching exon skipping. **(C)** Exonic retention. Chevron side indicates partial codon. SR, serine-arginine rich splicing factors; hnRNP, heterogeneous ribonucleoprotein particle. ASO is shown in red or indicated ASO in the figure. Created with BioRender.com.

The earliest and most commonly applied ASO-mediated mode of action was RNase-H mediated cleavage (Crooke et al., 2021). RNase-H is essential for gene stability and most notably is used to cleave RNA primers in Okazaki fragments involved in DNA replication (Cerritelli and Crouch, 2009). Additionally, it plays a cooperative role in the prevention of R-loop accumulation which induce genome instability as a result of transcription-induced supercoiling, a hallmark of cancer cells (Broccoli et al., 2004; Santos-Pereira and Aguilera, 2015). These proteins are grouped into two distinct categories based on their substrates for enzyme cleavage. RNase-H1 can function independently of the cell cycle and cleaves the phosphodiester bonds of RNA in RNA:DNA hybrids. While RNase-H2 has strict cell-cycle requirements and plays a similar role with the addition of cleaving the single ribonucleotides embedded within DNA. RNase-H1 based cleavage is the mechanism by which many partially modified PS-ASOs operate. Once the ASO hybridises and forms a duplex with the pre-mRNA/mRNA, the RNase-H1 cleaves the RNA target, exposing the transcript to exonuclease action to accelerate degradation, resulting in the downregulation of specific gene expression (Dias and Stein, 2002). This is outlined in Figure 6A.

#### 4.1.2 Occupancy-only (steric hindrance)

Steric hindrance offers several pathways for manipulating gene expression, these include influencing translation, splicing and polyadenylation. However, many of these methods fall outside the scope of our review. One of the greatest applications of steric hindrance in human therapeutic settings involves splice switching, which can degrade/restore reading frames and downregulate/upregulate gene and protein expression. This mechanism works through designing ASOs that anneal to complementary sequences within or flanking an exon or intron. The ASO then blocks regions critical to the delicate balance of exon:intron recognition by the spliceosome, resulting in the region being excised or retained in the mature mRNA. If the ASO targets positive splicing motifs of pre-mRNA, then this should typically induce exon skipping via inhibiting recognition of the exon by the spliceosome. Conversely, an ASO can be designed to target silencer motifs in pre-mRNA, which will result in a retention of the sequence in the mature mRNA. This mechanism essentially modifies the pre-mRNA’s usual splicing machinery via altering the recognition of the natural or cryptic splice sites by the spliceosome, (Figures 6B,C).

One such mechanism with potential to treat DS is exon skipping, which can alter the expression of the subsequent transcript dependent on the type of exon that is targeted. If an exon is targeted for excision and if that retains the reading frame, then this will result in the formation of a truncated and potentially functional protein, like that seen in the treatment for Duchenne muscular dystrophy and *ATXN3* (Mcclorey et al., 2006; Moore et al., 2017; Mcintosh et al., 2019). In contrast, targeting an exon that, if excised, induced a disruption in the reading frame would result in a non-functional protein that would be degraded via nonsense-mediated decay. This approach is similar to the treatment for multiple sclerosis, which targets *ITGA4* (Aung-Htut et al., 2019). Consequences of a disrupted reading frame can be seen in Figure 6C. Additionally, mechanisms such as isoform switching or translation blocking could be utilised, however, these are outside the scope of the current review.

### 4.2 FDA approved ASOs

As of April 2023, there have been a total of 13 antisense oligonucleotide therapies approved by the FDA, outlined in Table 3. These have received approval to treat previously untreatable genetic-based diseases like spinal muscular atrophy, Duchenne muscular dystrophy and familial amyloid polyneuropathy (Shadid et al., 2021). In this review we will not spend the time to explore the various FDA approved ASOs as this is outside the scope.

**Table 3:** Summary of currently available ASO therapies that achieved FDA approval.

Drug name	Brand name	Disease	ASO Mechanism	ASO Chemistry	Approval year	References
Fomivirsen	Vitravene^®^	CMV retinitis	RNase-H	PS-based	1998	de Smet et al. (1999)
Mipomersen	Kynamro^®^	Familial hyper-cholesterolemia	RNase-H	PS-based	2013	Thomas et al. (2013)
Eteplirsen	Exondys 51^™^	Duchenne Muscular Dystrophy	Exon Skipping	PMO	2016	Eser and Topaloğlu (2022)
Nusinersen	Spinraza^®^	Spinal muscular atrophy	Exon Inclusion	PS-based	2016	Goodkey et al. (2018)
Defibrotide	Defitelio^®^	Veno-occlusive disease	Complex	Phospho-diester Backbone	2016	Aziz et al. (2018)
Milasen	N/A	Batten disease	Exon skipping	2’-MOE	2017	Aartsma-Rus et al. (2023)
Inotersen	Tegsedi^®^	Transthyretin-mediated amyloidosis	RNase-H	2’-MOE	2018	Mathew and Wang (2019)
Golodirsen	Vyondys 53^™^	Duchenne Muscular Dystrophy	Exon Skipping	PMO	2019	Eser and Topaloğlu (2022)
Viltolarsen	Viltepso^®^	Duchenne Muscular Dystrophy	Exon Skipping	PMO	2020	Patterson et al. (2023)
Casimersen	Amondys 45^™^	Duchenne Muscular Dystrophy	Exon Skipping	PMO	2021	Wilton-Clark and Yokota (2021)
Tofersen	QALSODY^™^	Amyloid Lateral Sclerosis	RNase-H	PS 2′-MOE	2023	van Roon-Mom et al. (2023)

ASO, antisense oligonucleotide; CMV, cytomegalovirus; FDA, US Food and Drug Administration; PMO, phosphorodiamidate morpholino oligomer; PS, phosphorothioate; MOE, 2-Methoxyethyl.

### 4.3 Benefits of ASOs and challenges of CNS delivery

Developing treatments for neurological conditions remain some of the most challenging but these conditions have become a major focus for researchers in the field of oligonucleotide therapy. Antisense oligonucleotide researchers and clinicians treating neurological disorders are faced with the ongoing challenges of CNS drug development. Currently, there are no effective modalities of reaching the CNS without invasive methods. For example, spinal muscular atrophy treatment has seen success using ASOs, however drug delivery involves an invasive injection into the spinal canal.

To provide some context, spinal muscular atrophy is the most common genetic cause of death in infants and is an inherited neurological disorder that leads to the atrophy of the alpha motor neurons (D'amico et al., 2011). This causes a degeneration of the bulbar and spinal muscles, in addition to respiratory muscles, which later result in respiratory failure. The 2′-Methoxyethyl (MOE) PS ASO Nusinersen was approved by the FDA in 2016. This ASO increased the amount of SMN protein via inhibition of the negative splicing factors of intron 7 in the *SMN2* pre-mRNA, thereby promoting the inclusion of exon 7 (Wurster and Ludolph, 2018). Phase I, II, and III studies on infants found statistically significant improvements after treatment with Nusinersen, which ultimately led the drug from the bench to the clinic (Finkel et al., 2016, 2017a; Darras et al., 2019). However, much like other ASOs, the treatment had to be administered repeatedly; on days 0, 15, 29, 64, and then every 4 months (Finkel et al., 2017b). Secondly, Nusinersen is too large a molecule to cross the BBB. Therefore, the ASO had to be administered via invasive intrathecal injection. The crossing of the BBB is one of the largest challenges facing drug treatment of neurological disorders.

**Figure 7:**
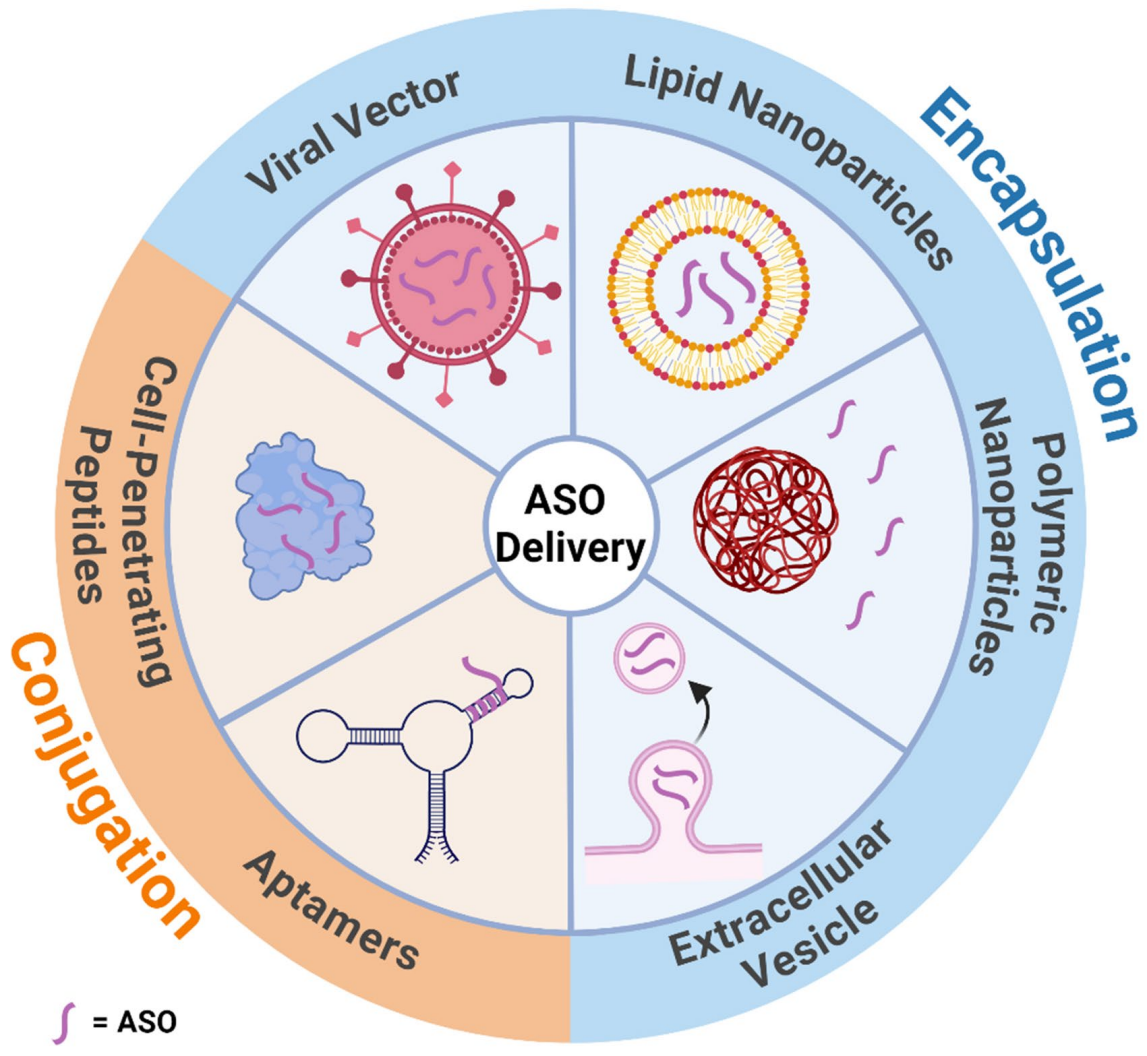
Illustration of the various mechanisms that can assist in antisense oligonucleotide delivery through the blood brain barrier. Delivery methods have been categorised by whether they encapsulate or are conjugated to the antisense oligonucleotide. Created with BioRender.com.

The BBB is the divider between the CNS and the periphery, acting as a mediator which protects the brain from toxic substances while allowing a steady supply of nutrients. Many advances in ASO chemistry have improved cellular uptake, although not many have been designed to overcome the crossing of the BBB. Current ASOs do not efficiently cross the BBB due to their charged nature and large size, with some studies finding that less than 1% of ASOs delivered peripherally reach the brain (Agrawal et al., 1991; Cossum et al., 1993; Banks et al., 2001; Farr et al., 2014). Multiple methods are being investigated to increase BBB penetrating efficiency. One method utilises receptor-mediated endocytosis which has been used to successfully deliver ASOs to the brains of parenchyma via nanoparticles (Pardridge, 2007; Kozlu et al., 2014). Another method utilised is a 5–30 amino acid long, positively-charged, cell-penetrating peptide which has shown successful distribution in the brain after crossing the BBB in mice (El-Andaloussi et al., 2005; Du et al., 2011). Adeno-associated viral vectors and encapsulating the gene therapies in exosomes have shown some promise in crossing the BBB (Stanimirovic et al., 2018). However, adeno-associated viral vectors have shown some levels of toxicity in recent studies on primates (Keiser et al., 2021). This leaves the only effective modality of delivery being direct administration to the CNS via intrathecal injection (Geary et al., 2015). While this has been an effective delivery method for many individuals, there is a risk of developing lumbar puncture syndrome (Cordts et al., 2020). A summary of the main delivery methods for ASOs can be found in Figure 7.

We have identified a few potential strategies to modulate the expression of *DYRK1A* through utilising ASOs. One possible method for addressing DS phenotypes would be to lower the expression of a dose-sensitive, over-expressed gene transcript via inducing exon skipping to disrupt the reading frame. Theoretically, this will result in nonsense-mediated decay of the transcript and thereby reduce the expression of the over-expressed target protein. For this treatment to be effective, one would need to be aware of the importance of dosing the ASO to not suppress *DYRK1A* expression in an excessive manner (most likely around 33% reduction—for gene dosage normalisation), as this could result in negative side effects that may appear similar to DYRK1A syndrome phenotypes. It is unlikely to be completely suppressed but there would need to be tests on optimising dosage using titrations which may prove challenging, but possible. Antisense oligonucleotides offer an exquisite method for specificity and sensitivity. The *DYRK* genes have enough genetic differences to design ASOs that only target *DYRK1A* (Aranda et al., 2011). Moreover, although various *DYRK1A* transcripts exist, all have a similar reading frame, thus targeting out of frame exons in one transcript should target all *DYRK1A* transcripts with a similar effect.

Ultimately, ASOs have a high potential to treat numerous neurological disorders with high specificity. With current advancements, particularly with the more recent PMO and peptide-conjugated PMO technologies, we have witnessed significant increases in the safety profile and efficacy. Ultimately, providing these an advantage when compared to other DYRK1A inhibitors. Current research is highly focused on applying this treatment strategy to many other disorders that have previously been deemed untreatable. Current research has attempted to utilise ASOs to treat myotonic dystrophies, Huntington’s disease, amyotrophic lateral sclerosis, and Alzheimer’s disease to name a few (Gao and Cooper, 2013; Hinrich et al., 2016; Aslesh and Yokota, 2020; Boros et al., 2022). As ASO therapies become more widely adopted, we should see a reduction in costs due to streamlining synthesis consistency and efficiency. Particularly, the possibility of class approval for ASOs will speed up the application process and reduce costs attributable to ongoing clinical safety trials (Aartsma-Rus and Krieg, 2017). Moreover, if ASOs can be applied to widespread disorders like DS, the increased demand should help distribute the cost, making them more affordable.

## 5 Conclusion

This literature review has outlined the great potential for ASOs to be used as a treatment for DS through downregulating *DYRK1A*. We aimed to distil current research perspectives on DS, *DYRK1A*, and ASOs to justify the potential of using ASOs to address the cognitive deficits associated with DS. Much of the literature supports that *DYRK1A* is a vital gene implicated in neural development, function and repair. Promising research that has normalised DYRK1A expression in DS animal models have shown improvements across numerous cognitive abilities. We have also shown that whilst numerous DYRK1A inhibitors are under investigation, all are aimed toward altering the protein’s function. Whereas a DYRK1A-targeted ASO would uniquely target the transcript directly, offering enhanced selectivity compared to the other inhibitors and thereby minimising off-target effects. While challenges persist in implementing ASO-based therapeutics, such as their difficult delivery to the CNS, recent advances in the field provide some hope. These include the development of delivery systems and improvements in specificity, potency, and stability. As a result, numerous ASOs have received FDA approval, further cementing their therapeutic potential for treating previously untreatable genetic-based disorders. In conclusion, ASOs targeting *DYRK1A* would not only aid in mitigating the cognitive deficits from DS but also have the potential to address a broader range of neurological and other diseases. As research and advancements continue, ASOs will become more refined and hopefully overcome the previously mentioned limitations. We are currently on the cusp of a future where individuals with DS can experience substantial improvements in their cognitive functioning and overall quality of life.

## Author contributions

AM: Writing – review & editing, Conceptualization, Data curation, Writing – original draft. SW: Supervision, Writing – review & editing. MA-H: Supervision, Writing – review & editing. CM: Funding acquisition, Resources, Supervision, Writing – review & editing.

## References

[ref1] Aartsma-Rus A. Garanto A. Van Roon-Mom W. Mcconnell E. M. Suslovitch V. Yan W. X.. (2023). Consensus guidelines for the design and in vitro preclinical efficacy testing N-of-1 exon skipping antisense oligonucleotides. Nucl. Acid Therapeut. 33, 17–25. doi: 10.1089/nat.2022.0060, PMID: 36516128 PMC9940807

[ref2] Aartsma-Rus A. Krieg A. M. (2017). Fda approves eteplirsen for Duchenne muscular dystrophy: the next chapter in the eteplirsen saga. Nucl. Acid Therapeut. 27, 1–3. doi: 10.1089/nat.2016.0657, PMID: 27929755 PMC5312460

[ref3] Abbeduto L. Pavetto M. Kesin E. Weissman M. Karadottir S. O’brien A.. (2001). The linguistic and cognitive profile of Down syndrome: evidence from a comparison with fragile X syndrome. Down Syndrome Res. Practice 7, 9–15. doi: 10.3104/reports.109, PMID: 11706811

[ref4] Adayev T. Wegiel J. Hwang Y.-W. (2011). Harmine is an Atp-competitive inhibitor for dual-specificity tyrosine phosphorylation-regulated kinase 1A (Dyrk1A). Arch. Biochem. Biophys. 507, 212–218. doi: 10.1016/j.abb.2010.12.024, PMID: 21185805 PMC3062630

[ref5] Agrawal S. Temsamani J. Tang J. Y. (1991). Pharmacokinetics, biodistribution, and stability of oligodeoxynucleotide phosphorothioates in mice. Proc. Natl. Acad. Sci. 88, 7595–7599. doi: 10.1073/pnas.88.17.7595 1881900 PMC52348

[ref6] Al-Biltagi M. (2015). Down syndrome from epidemiologic point of view. Ec Paediatrics 2, 82–91.

[ref7] Alexeeva M. Åberg E. Engh R. A. Rothweiler U. (2015). The structure of a dual-specificity tyrosine phosphorylation-regulated kinase 1A-Pkc412 complex reveals disulfide-bridge formation with the anomalous catalytic loop Hrd (Hcd) cysteine. Acta Crystallogr. D Biol. Crystallogr. 71, 1207–1215. doi: 10.1107/S1399004715005106, PMID: 25945585

[ref8] Amantana A. Moulton H. M. Cate M. L. Reddy M. T. Whitehead T. Hassinger J. N.. (2007). Pharmacokinetics, biodistribution, stability and toxicity of a cell-penetrating peptide− morpholino oligomer conjugate. Bioconjug. Chem. 18, 1325–1331. doi: 10.1021/bc070060v, PMID: 17583927

[ref9] Ananthapadmanabhan V. Shows K. H. Dickinson A. J. Litovchick L. (2023). Insights from the protein interaction universe of the multifunctional “goldilocks” kinase Dyrk1A. Front. Cell Dev. Biol. 11:277537. doi: 10.3389/fcell.2023.1277537, PMID: 37900285 PMC10600473

[ref10] Anguela X. M. High K. A. (2019). Entering the modern era of gene therapy. Annu. Rev. Med. 70, 273–288. doi: 10.1146/annurev-med-012017-043332, PMID: 30477394

[ref11] Anneren G. Edman B. (1993). Down syndrome--a gene dosage disease caused by trisomy of genes within a small segment of the long arm of chromosome 21, exemplified by the study of effects from the superoxide-dismutase type 1 (SOD-1) gene. APMIS Suppl. 40, 71–79, PMID: 8311993

[ref12] Araldi G. L. Hwang Y.-W. (2022). Design, synthesis, and biological evaluation of polyphenol derivatives as Dyrk1A inhibitors. The discovery of a potentially promising treatment for multiple sclerosis. Bioorg. Med. Chem. Lett. 64:128675. doi: 10.1016/j.bmcl.2022.128675, PMID: 35292341 PMC9067539

[ref13] Aranda S. Laguna A. Luna S. D. L. (2011). Dyrk family of protein kinases: evolutionary relationships, biochemical properties, and functional roles. FASEB J. 25, 449–462. doi: 10.1096/fj.10-165837, PMID: 21048044

[ref14] Arbones M. L. Thomazeau A. Nakano-Kobayashi A. Hagiwara M. Delabar J. M. (2019). Dyrk1A and cognition: a lifelong relationship. Pharmacol. Ther. 194, 199–221. doi: 10.1016/j.pharmthera.2018.09.010, PMID: 30268771

[ref15] Arque G. Casanovas A. Dierssen M. (2013). Dyrk1A is dynamically expressed on subsets of motor neurons and in the neuromuscular junction: possible role in Down syndrome. PLoS One 8:e54285. doi: 10.1371/journal.pone.0054285, PMID: 23342120 PMC3546979

[ref16] Aslesh T. Yokota T. (2020). Development of antisense oligonucleotide gapmers for the treatment of Huntington’s disease. Gapmers: Methods and Protocols, 57–67. 10.1007/978-1-0716-0771-8_4 32865782

[ref17] Atas-Ozcan H. Brault V. Duchon A. Herault Y. (2021). Dyrk1a from gene function in development and physiology to dosage correction across life span in down syndrome. Genes 12:1833. doi: 10.3390/genes12111833, PMID: 34828439 PMC8624927

[ref18] Aung-Htut M. T. Comerford I. Johnsen R. Foyle K. Fletcher S. Wilton S. D. (2019). Reduction of integrin alpha 4 activity through splice modulating antisense oligonucleotides. Sci. Rep. 9:12994. doi: 10.1038/s41598-019-49385-6, PMID: 31506448 PMC6736852

[ref19] Aziz M. T. Kakadiya P. P. Kush S. M. Weigel K. Lowe D. K. (2018). Defibrotide: an oligonucleotide for sinusoidal obstruction syndrome. Ann. Pharmacother. 52, 166–174. doi: 10.1177/1060028017732586, PMID: 28914546

[ref20] Bain J. Plater L. Elliott M. Shpiro N. Hastie C. J. Mclauchlan H.. (2007). The selectivity of protein kinase inhibitors: a further update. Biochem. J. 408, 297–315. doi: 10.1042/BJ20070797, PMID: 17850214 PMC2267365

[ref21] Ballard C. Mobley W. Hardy J. Williams G. Corbett A. (2016). Dementia in Down’s syndrome. Lancet Neurol. 15, 622–636. doi: 10.1016/S1474-4422(16)00063-6 27302127

[ref22] Banks W. A. Farr S. A. Butt W. Kumar V. B. Franko M. W. Morley J. E. (2001). Delivery across the blood-brain barrier of antisense directed against amyloid β: reversal of learning and memory deficits in mice overexpressing amyloid precursor protein. J. Pharmacol. Exp. Ther. 297, 1113–1121, PMID: 11356936

[ref23] Baruchel A. Bourquin J.-P. Crispino J. Cuartero S. Hasle H. Hitzler J.. (2023). Down syndrome and leukemia: from basic mechanisms to clinical advances. Haematologica 108, 2570–2581. doi: 10.3324/haematol.2023.283225, PMID: 37439336 PMC10542835

[ref24] Baxter L. L. Moran T. H. Richtsmeier J. T. Troncoso J. Reeves R. H. (2000). Discovery and genetic localization of Down syndrome cerebellar phenotypes using the Ts65Dn mouse. Hum. Mol. Genet. 9, 195–202. doi: 10.1093/hmg/9.2.195, PMID: 10607830

[ref25] Becker W. Soppa U. Tejedor J. (2014). Dyrk1A: a potential drug target for multiple Down syndrome neuropathologies. CNS Neurol Disorders Drug Targets 13, 26–33. doi: 10.2174/18715273113126660186 24152332

[ref26] Becker W. Weber Y. Wetzel K. Eirmbter K. Tejedor F. J. Joost H.-G. (1998). Sequence characteristics, subcellular localization, and substrate specificity of Dyrk-related kinases, a novel family of dual specificity protein kinases. J. Biol. Chem. 273, 25893–25902. doi: 10.1074/jbc.273.40.25893, PMID: 9748265

[ref27] Belichenko N. P. Belichenko P. V. Kleschevnikov A. M. Salehi A. Reeves R. H. Mobley W. C. (2009). The “Down syndrome critical region” is sufficient in the mouse model to confer behavioral, neurophysiological, and synaptic phenotypes characteristic of Down syndrome. J. Neurosci. 29, 5938–5948. doi: 10.1523/JNEUROSCI.1547-09.2009, PMID: 19420260 PMC3849469

[ref28] Beniddir M. A. Le Borgne E. Iorga B. I. LoaëC N. G. Lozach O. Meijer L.. (2014). Acridone alkaloids from Glycosmis chlorosperma as Dyrk1A inhibitors. J. Nat. Prod. 77, 1117–1122. doi: 10.1021/np400856h, PMID: 24798019

[ref29] Bornstein E. Lenchner E. Donnenfeld A. Jodicke C. Keeler S. M. Kapp S.. (2010). Complete trisomy 21 vs translocation Down syndrome: a comparison of modes of ascertainment. Am. J. Obstet. Gynecol. 203–391, e1–e391. doi: 10.1016/j.ajog.2010.06.019 20691415

[ref30] Boros B. D. Schoch K. M. Kreple C. J. Miller T. M. (2022). Antisense oligonucleotides for the study and treatment of Als. Neurotherapeutics 19, 1145–1158. doi: 10.1007/s13311-022-01247-2, PMID: 35653060 PMC9587169

[ref31] Broccoli S. Rallu F. Sanscartier P. Cerritelli S. M. Crouch R. J. Drolet M. (2004). Effects of Rna polymerase modifications on transcription-induced negative supercoiling and associated R-loop formation. Mol. Microbiol. 52, 1769–1779. doi: 10.1111/j.1365-2958.2004.04092.x, PMID: 15186424

[ref32] Bronicki L. M. Redin C. Drunat S. Piton A. Lyons M. Passemard S.. (2015). Ten new cases further delineate the syndromic intellectual disability phenotype caused by mutations in Dyrk1A. Eur. J. Hum. Genet. 23, 1482–1487. doi: 10.1038/ejhg.2015.29, PMID: 25920557 PMC4613470

[ref33] Bruel A. Bénéteau R. Chabanne M. Lozach O. Le Guevel R. Ravache M.. (2014). Synthesis of new pyridazino [4, 5-b] indol-4-ones and pyridazin-3 (2H)-one analogs as Dyrk1A inhibitors. Bioorg. Med. Chem. Lett. 24, 5037–5040. doi: 10.1016/j.bmcl.2014.09.017, PMID: 25248682

[ref34] Bull M. J. Genetics C. O. (2011). Health supervision for children with Down syndrome, vol. 128. Il, USA: American Academy of Pediatrics Elk Grove Village, 393–406. 10.1542/peds.2011-1605 21788214

[ref35] Calabrese M. Rinaldi F. Grossi P. Gallo P. (2011). Cortical pathology and cognitive impairment in multiple sclerosis. Expert. Rev. Neurother. 11, 425–432. doi: 10.1586/ern.10.155 21375447

[ref36] Carlesimo G. A. Marotta L. Vicari S. (1997). Long-term memory in mental retardation: evidence for a specific impairment in subjects with Down's syndrome. Neuropsychologia 35, 71–79. doi: 10.1016/S0028-3932(96)00055-3, PMID: 8981379

[ref37] Carr J. (1988). Six weeks to twenty-one years old: a longitudinal study of children with Down's syndrome and their families: third Jack Tizard memorial lecture. J. Child Psychol. Psychiatry 29, 407–431. doi: 10.1111/j.1469-7610.1988.tb00734.x, PMID: 2975288

[ref38] Cerritelli S. M. Crouch R. J. (2009). Ribonuclease H: the enzymes in eukaryotes. FEBS J. 276, 1494–1505. doi: 10.1111/j.1742-4658.2009.06908.x 19228196 PMC2746905

[ref39] Chakrabarti L. Galdzicki Z. Haydar T. F. (2007). Defects in embryonic neurogenesis and initial synapse formation in the forebrain of the Ts65Dn mouse model of Down syndrome. J. Neurosci. 27, 11483–11495. doi: 10.1523/JNEUROSCI.3406-07.2007, PMID: 17959791 PMC6673208

[ref40] Channell M. M. Thurman A. J. Kover S. T. Abbeduto L. (2014). Patterns of change in nonverbal cognition in adolescents with Down syndrome. Res. Dev. Disabil. 35, 2933–2941. doi: 10.1016/j.ridd.2014.07.014, PMID: 25112795 PMC4155014

[ref41] Chapman R. (2006). Language learning in Down syndrome: the speech and language profile compared to adolescents with cognitive impairment of unknown origin. Down Syndrome Res. Practice 10, 61–66. doi: 10.3104/reports.306, PMID: 16869363

[ref42] Chapman R. S. Hesketh L. J. (2000). Behavioral phenotype of individuals with Down syndrome. Ment. Retard. Dev. Disabil. Res. Rev. 6, 84–95. doi: 10.1002/1098-2779(2000)6:2<84::AID-MRDD2>3.0.CO;2-P 10899801

[ref43] Contestabile A. Benfenati F. Gasparini L. (2010). Communication breaks-Down: from neurodevelopment defects to cognitive disabilities in Down syndrome. Prog. Neurobiol. 91, 1–22. doi: 10.1016/j.pneurobio.2010.01.003, PMID: 20097253

[ref44] Coppedè F. (2016). Risk factors for Down syndrome. Arch. Toxicol. 90, 2917–2929. doi: 10.1007/s00204-016-1843-3 27600794

[ref45] Cordts I. Lingor P. Friedrich B. Pernpeintner V. Zimmer C. Deschauer M.. (2020). Intrathecal nusinersen administration in adult spinal muscular atrophy patients with complex spinal anatomy. Ther. Adv. Neurol. Disord. 13:1756286419887616. doi: 10.1177/1756286419887616 32010224 PMC6974755

[ref46] Cossum P. A. Sasmor H. Dellinger D. Truong L. Cummins L. Owens S. R.. (1993). Disposition of the 14C-labeled phosphorothioate oligonucleotide Isis 2105 after intravenous administration to rats. J. Pharmacol. Exp. Ther. 267, 1181–1190, PMID: 8166890

[ref47] Courcet J.-B. Faivre L. Malzac P. Masurel-Paulet A. Lopez E. Callier P.. (2012). The Dyrk1A gene is a cause of syndromic intellectual disability with severe microcephaly and epilepsy. J. Med. Genet. 49, 731–736. doi: 10.1136/jmedgenet-2012-101251 23099646

[ref48] Couzens D. Cuskelly M. Haynes M. (2011). Cognitive development and Down syndrome: age-related change on the Stanford-Binet test. Am. J. Intellect. Dev. Disabil. 116, 181–204. doi: 10.1352/1944-7558-116.3.181, PMID: 21591843

[ref49] Crooke S. T. Baker B. F. Crooke R. M. Liang X.-H. (2021). Antisense technology: an overview and prospectus. Nat. Rev. Drug Discov. 20, 427–453. doi: 10.1038/s41573-021-00162-z, PMID: 33762737

[ref50] D'amico A. Mercuri E. Tiziano F. D. Bertini E. (2011). Spinal muscular atrophy. Orphanet J. Rare Dis. 6, 1–10. doi: 10.1186/1750-1172-6-71 22047105 PMC3231874

[ref51] Darras B. T. Chiriboga C. A. Iannaccone S. T. Swoboda K. J. Montes J. Mignon L.. (2019). Nusinersen in later-onset spinal muscular atrophy: long-term results from the phase 1/2 studies. Neurology 92, e2492–e2506. doi: 10.1212/WNL.0000000000007527, PMID: 31019106 PMC6541434

[ref52] De La Torre R. De Sola S. Hernandez G. Farré M. Pujol J. Rodriguez J.. (2016). Safety and efficacy of cognitive training plus epigallocatechin-3-gallate in young adults with Down's syndrome (Tesdad): a double-blind, randomised, placebo-controlled, phase 2 trial. The Lancet Neurology 15, 801–810. doi: 10.1016/S1474-4422(16)30034-5, PMID: 27302362

[ref53] De La Torre R. De Sola S. Pons M. Duchon A. De Lagran M. M. Farré M.. (2014). Epigallocatechin-3-gallate, a Dyrk1A inhibitor, rescues cognitive deficits in D own syndrome mouse models and in humans. Mol. Nutr. Food Res. 58, 278–288. doi: 10.1002/mnfr.201300325, PMID: 24039182

[ref54] De Smet M. D. Meenken C. Van Den Horn G. J. (1999). Fomivirsen–a phosphorothioate oligonucleotide for the treatment of cmv retinitis. Ocul. Immunol. Inflamm. 7, 189–198. doi: 10.1076/ocii.7.3.189.4007, PMID: 10611727

[ref55] Deboever E. Fistrovich A. Hulme C. Dunckley T. (2022). The omnipresence of Dyrk1A in human diseases. Int. J. Mol. Sci. 23:9355. doi: 10.3390/ijms23169355, PMID: 36012629 PMC9408930

[ref56] Delabar J.-M. Theophile D. Rahmani Z. Chettouh Z. Blouin J.-L. Prieur M.. (1993). Molecular mapping of twenty-four features of Down syndrome on chromosome 21. Eur. J. Hum. Genet. 1, 114–124. doi: 10.1159/000472398 8055322

[ref57] Deleavey G. F. Damha M. J. (2012). Designing chemically modified oligonucleotides for targeted gene silencing. Chem. Biol. 19, 937–954. doi: 10.1016/j.chembiol.2012.07.011 22921062

[ref58] Demange L. Abdellah F. N. Lozach O. Ferandin Y. Gresh N. Meijer L.. (2013). Potent inhibitors of Cdk5 derived from roscovitine: synthesis, biological evaluation and molecular modelling. Bioorg. Med. Chem. Lett. 23, 125–131. doi: 10.1016/j.bmcl.2012.10.141 23218601

[ref59] Dias N. Stein C. (2002). Antisense oligonucleotides: basic concepts and mechanisms. Mol. Cancer Ther. 1, 347–355, PMID: 12489851

[ref60] Dierssen M. Ramakers G. J. (2006). Dendritic pathology in mental retardation: from molecular genetics to neurobiology. Genes Brain Behav. 5, 48–60. doi: 10.1111/j.1601-183X.2006.00224.x 16681800

[ref61] Dowjat W. K. Adayev T. Kuchna I. Nowicki K. Palminiello S. Hwang Y. W.. (2007). Trisomy-driven overexpression of Dyrk1A kinase in the brain of subjects with Down syndrome. Neurosci. Lett. 413, 77–81. doi: 10.1016/j.neulet.2006.11.026, PMID: 17145134 PMC1890010

[ref62] Down J. L. (1887). On some of the mental affections of childhood and youth, vol. 1. J. & A: Churchill, 256–259. 10.1136/bmj.1.1362.256 PMC2534114 20751780

[ref63] Du L. Kayali R. Bertoni C. Fike F. Hu H. Iversen P. L.. (2011). Arginine-rich cell-penetrating peptide dramatically enhances Amo-mediated Atm aberrant splicing correction and enables delivery to brain and cerebellum. Hum. Mol. Genet. 20, 3151–3160. doi: 10.1093/hmg/ddr217, PMID: 21576124 PMC3140820

[ref64] Duchon A. Herault Y. (2016). Dyrk1A, a dosage-sensitive gene involved in neurodevelopmental disorders, is a target for drug development in Down syndrome. Front. Behav. Neurosci. 104. doi: 10.3389/fnbeh.2016.00104 PMC4891327 27375444

[ref65] El-Andaloussi S. Holm T. Langel U. (2005). Cell-penetrating peptides: mechanisms and applications. Curr. Pharm. Des. 11, 3597–3611. doi: 10.2174/138161205774580796, PMID: 16305497

[ref66] Eser G. Topaloğlu H. (2022). Current outline of exon skipping trials in Duchenne muscular dystrophy. Genes 13:1241. doi: 10.3390/genes13071241, PMID: 35886024 PMC9320322

[ref67] Falke H. Chaikuad A. Becker A. Loaëc N. Lozach O. Abu Jhaisha S.. (2015). 10-Iodo-11 H-indolo [3, 2-c] quinoline-6-carboxylic acids are selective inhibitors of Dyrk1A. J. Med. Chem. 58, 3131–3143. doi: 10.1021/jm501994d, PMID: 25730262 PMC4506206

[ref68] Farr S. A. Erickson M. A. Niehoff M. L. Banks W. A. Morley J. E. (2014). Central and peripheral administration of antisense oligonucleotide targeting amyloid-β protein precursor improves learning and memory and reduces neuroinflammatory cytokines in Tg2576 (Aβppswe) mice. J. Alzheimers Dis. 40, 1005–1016. doi: 10.3233/JAD-131883, PMID: 24577464 PMC4136536

[ref69] Feki A. Hibaoui Y. (2018). Dyrk1A protein, a promising therapeutic target to improve cognitive deficits in Down syndrome. Brain Sci. 8:187. doi: 10.3390/brainsci8100187, PMID: 30332747 PMC6210095

[ref70] Ferraiuolo L. Heath P. R. Holden H. Kasher P. Kirby J. Shaw P. J. (2007). Microarray analysis of the cellular pathways involved in the adaptation to and progression of motor neuron injury in the Sod1 G93A mouse model of familial Als. J. Neurosci. 27, 9201–9219. doi: 10.1523/JNEUROSCI.1470-07.2007, PMID: 17715356 PMC6672214

[ref71] Fidler D. Most D. Philofsky A., (2008). The Down syndrome behavioural phenotype: Taking a developmental approach.

[ref72] Finkel R. S. Chiriboga C. A. Vajsar J. Day J. W. Montes J. De Vivo D. C.. (2016). Treatment of infantile-onset spinal muscular atrophy with nusinersen: a phase 2, open-label, dose-escalation study. Lancet 388, 3017–3026. doi: 10.1016/S0140-6736(16)31408-8, PMID: 27939059

[ref73] Finkel R. Kuntz N. Mercuri E. Chiriboga C. Darras B. Topaloglu H.. (2017a). Efficacy and safety of nusinersen in infants with spinal muscular atrophy (Sma): final results from the phase 3 endear study. Eur. J. Paediatr. Neurol. 21, e14–e15. doi: 10.1016/j.ejpn.2017.04.1219

[ref74] Finkel R. S. Mercuri E. Darras B. T. Connolly A. M. Kuntz N. L. Kirschner J.. (2017b). Nusinersen versus sham control in infantile-onset spinal muscular atrophy. N. Engl. J. Med. 377, 1723–1732. doi: 10.1056/NEJMoa1702752, PMID: 29091570

[ref75] Fitzpatrick K. Tuffnell D. Kurinczuk J. Knight M. (2017). Pregnancy at very advanced maternal age: a UK population-based cohort study. BJOG: an international. J. Obstetr. Gynaecol. 124, 1097–1106. doi: 10.1111/1471-0528.14269 PMC5484369 27581343

[ref76] Foster D. J. Brown C. R. Shaikh S. Trapp C. Schlegel M. K. Qian K.. (2018). Advanced sirna designs further improve in vivo performance of Galnac-sirna conjugates. Mol. Ther. 26, 708–717. doi: 10.1016/j.ymthe.2017.12.021, PMID: 29456020 PMC5910670

[ref77] Fotaki V. Dierssen M. Alcántara S. Martínez S. Martí E. Casas C.. (2002). Dyrk1A haploinsufficiency affects viability and causes developmental delay and abnormal brain morphology in mice. Mol. Cell. Biol. 22, 6636–6647. doi: 10.1128/MCB.22.18.6636-6647.2002, PMID: 12192061 PMC135639

[ref78] Foucourt A. Hédou D. Dubouilh-Benard C. Désiré L. Casagrande A.-S. Leblond B.. (2014). Design and synthesis of thiazolo [5, 4-f] quinazolines as Dyrk1A inhibitors, part I. Molecules 19, 15546–15571. doi: 10.3390/molecules191015546, PMID: 25268714 PMC6270991

[ref79] Frost D. Meechoovet B. Wang T. Gately S. Giorgetti M. Shcherbakova I.. (2011). β-Carboline compounds, including harmine, inhibit Dyrk1A and tau phosphorylation at multiple Alzheimer's disease-related sites. PLoS One 6:e19264. doi: 10.1371/journal.pone.0019264 21573099 PMC3089604

[ref80] Gao Z. Cooper T. A. (2013). Antisense oligonucleotides: rising stars in eliminating Rna toxicity in myotonic dystrophy. Hum. Gene Ther. 24, 499–507. doi: 10.1089/hum.2012.212, PMID: 23252746 PMC3655630

[ref81] García-Cerro S. Martínez P. Vidal V. Corrales A. Flórez J. Vidal R.. (2014). Overexpression of Dyrk1A is implicated in several cognitive, electrophysiological and neuromorphological alterations found in a mouse model of Down syndrome. PLoS One 9:e106572. doi: 10.1371/journal.pone.0106572, PMID: 25188425 PMC4154723

[ref82] Geary R. S. Norris D. Yu R. Bennett C. F. (2015). Pharmacokinetics, biodistribution and cell uptake of antisense oligonucleotides. Adv. Drug Deliv. Rev. 87, 46–51. doi: 10.1016/j.addr.2015.01.008, PMID: 25666165

[ref83] Gómez D. Solsona E. Guitart M. Baena N. Gabau E. Egozcue J.. (2000). Origin of trisomy 21 in Down syndrome cases from a Spanish population registry. Annales de genetique, 2000. Elsevier, 23–28. 10.1016/s0003-3995(00)00017-4 10818217

[ref84] Goodkey K. Aslesh T. Maruyama R. Yokota T., (2018). Nusinersen in the treatment of spinal muscular atrophy. Exon skipping and inclusion therapies: Methods and protocols, 69–76. 10.1007/978-1-4939-8651-4_4 30171535

[ref85] Gourdain S. Dairou J. Denhez C. Bui L. C. Rodrigues-Lima F. Janel N.. (2013). Development of Dandys, new 3, 5-diaryl-7-azaindoles demonstrating potent Dyrk1A kinase inhibitory activity. J. Med. Chem. 56, 9569–9585. doi: 10.1021/jm401049v, PMID: 24188002

[ref86] Grieco J. Pulsifer M. Seligsohn K. Skotko B. Schwartz A. (2015). Down syndrome: cognitive and behavioral functioning across the lifespan. American journal of medical Genetics part C: seminars in medical genetics. Wiley Online Library, 169, 135–149. 10.1002/ajmg.c.31439 25989505

[ref87] Grygier P. Pustelny K. Nowak J. Golik P. Popowicz G. M. Plettenburg O.. (2023). Silmitasertib (Cx-4945), a clinically used Ck2-kinase inhibitor with additional effects on Gsk3β and Dyrk1A kinases: a structural perspective. J. Med. Chem. 66, 4009–4024. doi: 10.1021/acs.jmedchem.2c01887, PMID: 36883902 PMC10041529

[ref88] Guard S. E. Poss Z. C. Ebmeier C. C. Pagratis M. Simpson H. Taatjes D. J.. (2019). The nuclear interactome of Dyrk1A reveals a functional role in Dna damage repair. Sci. Rep. 9:6539. doi: 10.1038/s41598-019-42990-5, PMID: 31024071 PMC6483993

[ref89] Guedj F. Pereira P. L. Najas S. Barallobre M.-J. Chabert C. Souchet B.. (2012). Dyrk1A: a master regulatory protein controlling brain growth. Neurobiol. Dis. 46, 190–203. doi: 10.1016/j.nbd.2012.01.007, PMID: 22293606

[ref90] Guedj F. Sébrié C. Rivals I. Ledru A. Paly E. Bizot J. C.. (2009). Green tea polyphenols rescue of brain defects induced by overexpression of Dyrk1A. PLoS One 4:e4606. doi: 10.1371/journal.pone.0004606, PMID: 19242551 PMC2645681

[ref91] Guimera J. Casas C. Estivill X. Pritchard M. (1999). Humanminibrainhomologue (mnbh/dyrk1): characterization, alternative splicing, differential tissue expression, and overexpression in down syndrome. Genomics 57, 407–418. doi: 10.1006/geno.1999.5775, PMID: 10329007

[ref92] Guralnick M. J. (2002). Involvement with peers: comparisons between young children with and without Down’s syndrome. J. Intellect. Disabil. Res. 46, 379–393. doi: 10.1046/j.1365-2788.2002.00405.x, PMID: 12031021

[ref93] Hasle H. Friedman J. M. Olsen J. H. Rasmussen S. A. (2016). Low risk of solid tumors in persons with Down syndrome. Genet. Med. 18, 1151–1157. doi: 10.1038/gim.2016.23, PMID: 27031084

[ref94] Hill D. A. Gridley G. Cnattingius S. Mellemkjaer L. Linet M. Adami H.-O.. (2003). Mortality and cancer incidence among individuals with Down syndrome. Arch. Intern. Med. 163, 705–711. doi: 10.1001/archinte.163.6.705 12639204

[ref95] Hinrich A. J. Jodelka F. M. Chang J. L. Brutman D. Bruno A. M. Briggs C. A.. (2016). Therapeutic correction of Apoer2 splicing in Alzheimer's disease mice using antisense oligonucleotides. EMBO Mol. Med. 8, 328–345. doi: 10.15252/emmm.201505846, PMID: 26902204 PMC4818756

[ref96] Hood J. Kc S. K. Wallace D. M. Mittapalli G. K. Hofilena B. J. Mak C. C.. (2017). 5-substituted indazole-3-carboxamides and preparation and use thereof. U.S. Patent 9,745,271.

[ref97] Hulme C. Dunckley T. Shaw Y.-J. (2020). Arizona Board of Regents of University of Arizona and Translational Genomics Research Institute TGen, 2020. Small molecule inhibitors of DYRK1A and uses thereof. U.S. Patent 10,730,842.

[ref98] Iversen P. (2001). Phosphorodiamidate morpholino oligomers: favorable properties for sequence-specific gene inactivation. Curr. Opin. Mol. Ther. 3, 235–238, PMID: 11497346

[ref99] Jack C. R. Lowe V. J. Senjem M. L. Weigand S. D. Kemp B. J. Shiung M. M.. (2008). 11C PiB and structural Mri provide complementary information in imaging of Alzheimer's disease and amnestic mild cognitive impairment. Brain 131, 665–680. doi: 10.1093/brain/awm336, PMID: 18263627 PMC2730157

[ref100] Jarhad D. B. Mashelkar K. K. Kim H.-R. Noh M. Jeong L. S. (2018). Dual-specificity tyrosine phosphorylation-regulated kinase 1A (Dyrk1A) inhibitors as potential therapeutics. J. Med. Chem. 61, 9791–9810. doi: 10.1021/acs.jmedchem.8b00185, PMID: 29985601

[ref101] Jiang X. Liu C. Yu T. Zhang L. Meng K. Xing Z.. (2015). Genetic dissection of the Down syndrome critical region. Hum. Mol. Genet. 24, 6540–6551. doi: 10.1093/hmg/ddv364, PMID: 26374847 PMC4614710

[ref102] Kaczmarski W. Barua M. Mazur-Kolecka B. Frackowiak J. Dowjat W. Mehta P.. (2014). Intracellular distribution of differentially phosphorylated dual-specificity tyrosine phosphorylation-regulated kinase 1A (Dyrk1A). J. Neurosci. Res. 92, 162–173. doi: 10.1002/jnr.23279, PMID: 24327345 PMC3951420

[ref103] Kava M. P. Tullu M. S. Muranjan M. N. Girisha K. (2004). Down syndrome clinical profile from India. Arch. Med. Res. 35, 31–35. doi: 10.1016/j.arcmed.2003.06.005 15036797

[ref104] Keiser M. S. Ranum P. T. Yrigollen C. M. Carrell E. M. Smith G. R. Muehlmatt A. L.. (2021). Toxicity after AAV delivery of RNAI expression constructs into nonhuman primate brain. Nat. Med. 27, 1982–1989. doi: 10.1038/s41591-021-01522-3, PMID: 34663988 PMC8605996

[ref105] Kemper T. L. (1991). Down syndrome. Normal and Altered States of Function: Springer.

[ref106] Kim H. Lee K.-S. Kim A.-K. Choi M. Choi K. Kang M.. (2016). A chemical with proven clinical safety rescues Down-syndrome-related phenotypes in through Dyrk1A inhibition. Dis. Model. Mech. 9, 839–848. doi: 10.1242/dmm.025668, PMID: 27483355 PMC5007978

[ref107] Korenberg J. R. Chen X. Schipper R. Sun Z. Gonsky R. Gerwehr S.. (1994). Down syndrome phenotypes: the consequences of chromosomal imbalance. Proc. Natl. Acad. Sci. 91, 4997–5001. doi: 10.1073/pnas.91.11.4997, PMID: 8197171 PMC43917

[ref108] Korenberg J. R. Kawashima H. Pulst S.-M. Ikeuchi T. Ogasawara N. Yamamoto K.. (1990). Molecular definition of a region of chromosome 21 that causes features of the Down syndrome phenotype. Am. J. Hum. Genet. 47, 236–246, PMID: 2143053 PMC1683719

[ref109] Kozlu S. Caban S. Yerlikaya F. Fernandez-Megia E. Novoa-Carballal R. Riguera R.. (2014). An aquaporin 4 antisense oligonucleotide loaded, brain targeted nanoparticulate system design. Int. J. Pharmaceut. Sci. 69, 340–345. 24855824

[ref110] Kurt M. A. Kafa M. I. Dierssen M. Davies D. C. (2004). Deficits of neuronal density in Ca1 and synaptic density in the dentate gyrus, Ca3 and Ca1, in a mouse model of Down syndrome. Brain Res. 1022, 101–109. doi: 10.1016/j.brainres.2004.06.075, PMID: 15353219

[ref111] Lamoral-Theys D. Pottier L. Dufrasne F. Neve J. Dubois J. Kornienko A.. (2010). Natural polyphenols that display anticancer properties through inhibition of kinase activity. Curr. Med. Chem. 17, 812–825. doi: 10.2174/092986710790712183, PMID: 20156174

[ref112] Lanfranchi S. Jerman O. Dal Pont E. Alberti A. Vianello R. (2010). Executive function in adolescents with Down syndrome. J. Intellect. Disabil. Res. 54, 308–319. doi: 10.1111/j.1365-2788.2010.01262.x 20202074

[ref113] Leder S. Weber Y. Altafaj X. Estivill X. Joost H.-G. Becker W. (1999). Cloning and characterization of Dyrk1B, a novel member of the Dyrk family of protein kinases. Biochem. Biophys. Res. Commun. 254, 474–479. doi: 10.1006/bbrc.1998.9967, PMID: 9918863

[ref114] Lefter S. Costello D. J. Mcnamara B. Sweeney B. (2011). Clinical and Eeg features of seizures in adults with down syndrome. J. Clin. Neurophysiol. 28, 469–473. doi: 10.1097/WNP.0b013e318230da76, PMID: 21946360

[ref115] Li J. J. Tian Y. L. Zhai H. L. Lv M. Zhang X. Y. (2016). Insights into mechanism of pyrido [2, 3-d] pyrimidines as Dyrk1A inhibitors based on molecular dynamic simulations. Proteins: structure. Function Bioinf 84, 1108–1123. doi: 10.1002/prot.25056 27119584

[ref116] Liang X.-H. Sun H. Shen W. Crooke S. T. (2015). Identification and characterization of intracellular proteins that bind oligonucleotides with phosphorothioate linkages. Nucleic Acids Res. 43, 2927–2945. doi: 10.1093/nar/gkv143, PMID: 25712094 PMC4357732

[ref117] Liu T. Wang Y. Wang J. Ren C. Chen H. Zhang J. (2022). Dyrk1A inhibitors for disease therapy: current status and perspectives. Eur. J. Med. Chem. 229:114062. doi: 10.1016/j.ejmech.2021.114062, PMID: 34954592

[ref118] Lochhead P. A. Sibbet G. Morrice N. Cleghon V. (2005). Activation-loop autophosphorylation is mediated by a novel transitional intermediate form of Dyrks. Cell 121, 925–936. doi: 10.1016/j.cell.2005.03.034, PMID: 15960979

[ref119] Lorenzi H. A. Reeves R. H. (2006). Hippocampal hypocellularity in the Ts65Dn mouse originates early in development. Brain Res. 1104, 153–159. doi: 10.1016/j.brainres.2006.05.022, PMID: 16828061

[ref120] Malt E. A. Dahl R. C. Haugsand T. M. Ulvestad I. H. Emilsen N. M. Hansen B.. (2013). Health and disease in adults with Down syndrome. Tidsskrift for Den norske legeforening. doi: 10.4045/tidsskr.12.0390 23381164

[ref121] Mathew V. Wang A. K. (2019). Inotersen: new promise for the treatment of hereditary transthyretin amyloidosis. Drug Des. Devel. Ther. 13, 1515–1525. doi: 10.2147/DDDT.S162913, PMID: 31118583 PMC6507904

[ref122] Matsumoto N. Ohashi H. Tsukahara M. Kim K. C. Soeda E. Niikawa N. (1997). Possible narrowed assignment of the loci of monosomy 21-associated microcephaly and intrauterine growth retardation to a 1.2-Mb segment at 21q22. 2. Am. J. Hum. Genet. 60, 997–999, PMID: 9106547 PMC1712454

[ref123] Mcclorey G. Moulton H. Iversen P. Fletcher S. Wilton S. (2006). Antisense oligonucleotide-induced exon skipping restores dystrophin expression in vitro in a canine model of Dmd. Gene Ther. 13, 1373–1381. doi: 10.1038/sj.gt.3302800, PMID: 16724091

[ref124] Mcdonald C. R. Gharapetian L. Mcevoy L. K. Fennema-Notestine C. Hagler D. J. Jr. Holland D.. (2012). Relationship between regional atrophy rates and cognitive decline in mild cognitive impairment. Neurobiol. Aging 33, 242–253. doi: 10.1016/j.neurobiolaging.2010.03.015, PMID: 20471718 PMC2923665

[ref125] Mcintosh C. S. Aung-Htut M. T. Fletcher S. Wilton S. D. (2019). Removal of the polyglutamine repeat of Ataxin-3 by redirecting pre-mrna processing. Int. J. Mol. Sci. 20:5434. doi: 10.3390/ijms20215434 31683630 PMC6862616

[ref126] Melton D. (1985). Injected anti-sense Rnas specifically block messenger Rna translation in vivo. Proc. Natl. Acad. Sci. 82, 144–148. doi: 10.1073/pnas.82.1.144, PMID: 3855537 PMC396988

[ref127] Moore L. R. Rajpal G. Dillingham I. T. Qutob M. Blumenstein K. G. Gattis D.. (2017). Evaluation of antisense oligonucleotides targeting Atxn3 in Sca3 mouse models. Molecular Therapy Nucl Acids 7, 200–210. doi: 10.1016/j.omtn.2017.04.005, PMID: 28624196 PMC5415970

[ref128] Moreau M. Benhaddou S. Dard R. Tolu S. Hamzé R. Vialard F.. (2021). Metabolic diseases and Down syndrome: how are they linked together? Biomedicines 9:221. doi: 10.3390/biomedicines9020221, PMID: 33671490 PMC7926648

[ref129] Muthumani P. (2020). Clinical profile of Down syndrome in a tertiary care Centre. Chennai: Madras Medical College.

[ref130] Naert G. Ferré V. Meunier J. Keller E. Malmström S. Givalois L.. (2015). Leucettine L41, a Dyrk1A-preferential Dyrks/Clks inhibitor, prevents memory impairments and neurotoxicity induced by oligomeric Aβ25–35 peptide administration in mice. Eur. Neuropsychopharmacol. 25, 2170–2182. doi: 10.1016/j.euroneuro.2015.03.018, PMID: 26381812

[ref131] Najas S. Arranz J. Lochhead P. A. Ashford A. L. Oxley D. Delabar J. M.. (2015). Dyrk1A-mediated cyclin D1 degradation in neural stem cells contributes to the neurogenic cortical defects in Down syndrome. EBioMedicine 2, 120–134. doi: 10.1016/j.ebiom.2015.01.010, PMID: 26137553 PMC4484814

[ref132] Nardo G. Trolese M. C. Tortarolo M. Vallarola A. Freschi M. Pasetto L.. (2016). New insights on the mechanisms of disease course variability in Als from mutant Sod1 mouse models. Brain Pathol. 26, 237–247. doi: 10.1111/bpa.12351, PMID: 26780365 PMC8029191

[ref133] Okui M. Ide T. Morita K. Funakoshi E. Ito F. Ogita K.. (1999). High-level expression of the Mnb/Dyrk1A gene in brain and heart during rat early development. Genomics 62, 165–171. doi: 10.1006/geno.1999.5998, PMID: 10610708

[ref134] Olson L. E. Richtsmeier J. T. Leszl J. Reeves R. H. (2004b). A chromosome 21 critical region does not cause specific Down syndrome phenotypes. Science 306, 687–690. doi: 10.1126/science.1098992, PMID: 15499018 PMC4019810

[ref135] Olson L. Roper R. Baxter L. Carlson E. Epstein C. Reeves R. H. (2004a). Down syndrome mouse models Ts65Dn, Ts1Cje, and Ms1Cje/Ts65Dn exhibit variable severity of cerebellar phenotypes. Dev. Dyn. Off. Publ. Am. Assoc. Anat. 230, 581–589. doi: 10.1002/dvdy.20079, PMID: 15188443

[ref136] Olson L. E. Roper R. J. Sengstaken C. L. Peterson E. A. Aquino V. Galdzicki Z.. (2007). Trisomy for the Down syndrome ‘critical region’is necessary but not sufficient for brain phenotypes of trisomic mice. Hum. Mol. Genet. 16, 774–782. doi: 10.1093/hmg/ddm022 17339268

[ref137] Papavassiliou P. Charalsawadi C. Rafferty K. Jackson-Cook C. (2015). Mosaicism for trisomy 21: a review. Am. J. Med. Genet. A 167, 26–39. doi: 10.1002/ajmg.a.36861 25412855

[ref138] Pardridge W. M. (2007). Blood–brain barrier delivery. Drug Discov. Today 12, 54–61. doi: 10.1016/j.drudis.2006.10.013 17198973

[ref139] Patterson G. Conner H. Groneman M. Blavo C. Parmar M. S. (2023). Duchenne muscular dystrophy: current treatment and emerging exon skipping and gene therapy approach. Eur. J. Pharmacol. 175675. doi: 10.1016/j.ejphar.2023.175675 36963652

[ref140] Pelleri M. C. Cicchini E. Locatelli C. Vitale L. Caracausi M. Piovesan A.. (2016). Systematic reanalysis of partial trisomy 21 cases with or without Down syndrome suggests a small region on 21q22. 13 as critical to the phenotype. Hum. Mol. Genet. 25, 2525–2538. doi: 10.1093/hmg/ddw116, PMID: 27106104 PMC5181629

[ref141] Pennington B. F. Moon J. Edgin J. Stedron J. Nadel L. (2003). The neuropsychology of Down syndrome: evidence for hippocampal dysfunction. Child Dev. 74, 75–93. doi: 10.1111/1467-8624.00522, PMID: 12625437

[ref142] Pérez-Cremades D. Hernández S. Blasco-Ibáñez J. M. Crespo C. Nacher J. Varea E. (2010). Alteration of inhibitory circuits in the somatosensory cortex of Ts65Dn mice, a model for Down’s syndrome. J. Neural Transm. 117, 445–455. doi: 10.1007/s00702-010-0376-9, PMID: 20157742

[ref143] Rachidi M. Lopes C. (2008). Mental retardation and associated neurological dysfunctions in Down syndrome: a consequence of dysregulation in critical chromosome 21 genes and associated molecular pathways. Eur. J. Paediatr. Neurol. 12, 168–182. doi: 10.1016/j.ejpn.2007.08.010, PMID: 17933568

[ref144] Rachidi M. Lopes C. (2011). “Mental retardation and human chromosome 21 gene overdosage: from functional genomics and molecular mechanisms towards prevention and treatment of the neuropathogenesis of Down syndrome” in Genomics, proteomics, and the nervous system (Springer).

[ref145] Ram G. Chinen J. (2011). Infections and immunodeficiency in Down syndrome. Clin. Exp. Immunol. 164, 9–16. doi: 10.1111/j.1365-2249.2011.04335.x, PMID: 21352207 PMC3074212

[ref146] Raveau M. Shimohata A. Amano K. Miyamoto H. Yamakawa K. (2018). Dyrk1A-haploinsufficiency in mice causes autistic-like features and febrile seizures. Neurobiol. Dis. 110, 180–191. doi: 10.1016/j.nbd.2017.12.003, PMID: 29223763

[ref147] Rothweiler U. Stensen W. Brandsdal B. O. Isaksson J. Leeson F. A. Engh R. A.. (2016). Probing the Atp-binding pocket of protein kinase Dyrk1A with benzothiazole fragment molecules. J. Med. Chem. 59, 9814–9824. doi: 10.1021/acs.jmedchem.6b01086, PMID: 27736065

[ref148] Ruaud L. Mignot C. Guët A. Ohl C. Nava C. Héron D.. (2015). Dyrk1A mutations in two unrelated patients. Eur. J. Med. Genet. 58, 168–174. doi: 10.1016/j.ejmg.2014.12.014, PMID: 25641759

[ref149] Ryoo S.-R. Jeong H. K. Radnaabazar C. Yoo J.-J. Cho H.-J. Lee H.-W.. (2007). Dyrk1A-mediated hyperphosphorylation of tau. J. Biol. Chem. 282, 34850–34857. doi: 10.1074/jbc.M707358200, PMID: 17906291

[ref150] Sacher F. Möller C. Bone W. Gottwald U. Fritsch M. (2007). The expression of the testis-specific Dyrk4 kinase is highly restricted to step 8 spermatids but is not required for male fertility in mice. Mol. Cell. Endocrinol. 267, 80–88. doi: 10.1016/j.mce.2006.12.041, PMID: 17292540

[ref151] Sánchez C. Salas A. P. Braña A. F. Palomino M. Pineda-Lucena A. Carbajo R. J.. (2009). Generation of potent and selective kinase inhibitors by combinatorial biosynthesis of glycosylated indolocarbazoles. Chem. Commun. 68, 4118–4120. doi: 10.1039/b905068j 19568652

[ref152] Santos-Pereira J. M. Aguilera A. (2015). R loops: new modulators of genome dynamics and function. Nat. Rev. Genet. 16, 583–597. doi: 10.1038/nrg3961 26370899

[ref153] Schimmel M. S. Hammerman C. Bromiker R. Berger I. (2006). Third ventricle enlargement among newborn infants with trisomy 21. Pediatrics 117, e928–e931. doi: 10.1542/peds.2005-1788, PMID: 16651295

[ref154] Schnabel F. Smogavec M. Funke R. Pauli S. Burfeind P. Bartels I. (2018). Down syndrome phenotype in a boy with a mosaic microduplication of chromosome 21q22. Mol. Cytogenet. 11, 1–5. doi: 10.1186/s13039-018-0410-4 30619508 PMC6310980

[ref155] Scoles D. R. Minikel E. V. Pulst S. M. (2019). Antisense oligonucleotides: a primer. Neurol. Genetics 5:e323. doi: 10.1212/NXG.0000000000000323 PMC6501637 31119194

[ref156] Setten R. L. Rossi J. J. Han S.-P. (2019). The current state and future directions of Rnai-based therapeutics. Nat. Rev. Drug Discov. 18, 421–446. doi: 10.1038/s41573-019-0017-4, PMID: 30846871

[ref157] Shadid M. Badawi M. Abulrob A. (2021). Antisense oligonucleotides: absorption, distribution, metabolism, and excretion. Expert Opin. Drug Metab. Toxicol. 17, 1281–1292. doi: 10.1080/17425255.2021.1992382, PMID: 34643122

[ref158] Silverman W. (2007). Down syndrome: cognitive phenotype. Ment. Retard. Dev. Disabil. Res. Rev. 13, 228–236. doi: 10.1002/mrdd.20156 17910084

[ref159] Singh B. N. Shankar S. Srivastava R. K. (2011). Green tea catechin, epigallocatechin-3-gallate (Egcg): mechanisms, perspectives and clinical applications. Biochem. Pharmacol. 82, 1807–1821. doi: 10.1016/j.bcp.2011.07.093, PMID: 21827739 PMC4082721

[ref160] Śmigielska-Kuzia J. Boćkowski L. Sobaniec W. Sendrowski K. Olchowik B. Cholewa M. (2011). A volumetric magnetic resonance imaging study of brain structures in children with Down syndrome. Neurol. Neurochir. Pol. 45, 363–369. doi: 10.1016/S0028-3843(14)60107-9, PMID: 22101997

[ref161] Soria-Pastor S. Gimenez M. Narberhaus A. Falcon C. Botet F. Bargallo N.. (2008). Patterns of cerebral white matter damage and cognitive impairment in adolescents born very preterm. Int. J. Dev. Neurosci. 26, 647–654. doi: 10.1016/j.ijdevneu.2008.08.001, PMID: 18765280

[ref162] Souchet B. Guedj F. Sahún I. Duchon A. Daubigney F. Badel A.. (2014). Excitation/inhibition balance and learning are modified by Dyrk1a gene dosage. Neurobiol. Dis. 69, 65–75. doi: 10.1016/j.nbd.2014.04.016, PMID: 24801365

[ref163] Soundararajan M. Roos A. K. Savitsky P. Filippakopoulos P. Kettenbach A. N. Olsen J. V.. (2013). Structures of Down syndrome kinases, Dyrks, reveal mechanisms of kinase activation and substrate recognition. Structure 21, 986–996. doi: 10.1016/j.str.2013.03.012, PMID: 23665168 PMC3677093

[ref164] Stanimirovic D. B. Sandhu J. K. Costain W. J. (2018). Emerging technologies for delivery of biotherapeutics and gene therapy across the blood–brain barrier. BioDrugs 32, 547–559. doi: 10.1007/s40259-018-0309-y, PMID: 30306341 PMC6290705

[ref165] Starbuck J. M. Llambrich S. Gonzàlez R. Albaigès J. Sarlé A. Wouters J.. (2021). Green tea extracts containing epigallocatechin-3-gallate modulate facial development in Down syndrome. Sci. Rep. 11, 1–13. doi: 10.1038/s41598-021-83757-1 33633179 PMC7907288

[ref166] Tazarki H. Zeinyeh W. Esvan Y. J. Knapp S. Chatterjee D. Schröder M.. (2019). New pyrido [3, 4-g] quinazoline derivatives as Clk1 and Dyrk1A inhibitors: synthesis, biological evaluation and binding mode analysis. Eur. J. Med. Chem. 166, 304–317. doi: 10.1016/j.ejmech.2019.01.052 30731399

[ref167] Tejedor F. J. Hämmerle B. (2011). Mnb/Dyrk1A as a multiple regulator of neuronal development. FEBS J. 278, 223–235. doi: 10.1111/j.1742-4658.2010.07954.x 21156027

[ref168] Tejedor F. Zhu X. Kaltenbach E. Ackermann A. Baumann A. Canal I.. (1995). Minibrain: a new protein kinase family involved in postembryonic neurogenesis in Drosophila. Neuron 14, 287–301. doi: 10.1016/0896-6273(95)90286-4, PMID: 7857639

[ref169] Thomas G. S. Cromwell W. C. Ali S. Chin W. Flaim J. D. Davidson M. (2013). Mipomersen, an apolipoprotein B synthesis inhibitor, reduces atherogenic lipoproteins in patients with severe hypercholesterolemia at high cardiovascular risk: a randomized, double-blind, placebo-controlled trial. J. Am. Coll. Cardiol. 62, 2178–2184. doi: 10.1016/j.jacc.2013.07.081, PMID: 24013058

[ref170] Thomazeau A. Lassalle O. Iafrati J. Souchet B. Guedj F. Janel N.. (2014). Prefrontal deficits in a murine model overexpressing the down syndrome candidate gene dyrk1a. J. Neurosci. 34, 1138–1147. doi: 10.1523/JNEUROSCI.2852-13.2014, PMID: 24453307 PMC3953590

[ref171] Torr J. Strydom A. Patti P. Jokinen N. (2010). Aging in Down syndrome: morbidity and mortality. J. Pol. Pract. Intellect. Disabil. 7, 70–81. doi: 10.1111/j.1741-1130.2010.00249.x

[ref172] Vacca R. A. Bawari S. Valenti D. Tewari D. Nabavi S. F. Shirooie S.. (2019). Down syndrome: neurobiological alterations and therapeutic targets. Neurosci. Biobehav. Rev. 98, 234–255. doi: 10.1016/j.neubiorev.2019.01.001, PMID: 30615933

[ref173] Van Bon B. W. Coe B. P. Bernier R. Green C. Gerdts J. Witherspoon K.. (2016). Disruptive de novo mutations of Dyrk1A lead to a syndromic form of autism and id. Mol. Psychiatry 21, 126–132. doi: 10.1038/mp.2015.5, PMID: 25707398 PMC4547916

[ref174] Van Bon B. W. Coe B. P. De Vries B. B. Eichler E. E., (2021). Dyrk1A syndrome. 26677511

[ref175] Van Roon-Mom W. Ferguson C. Aartsma-Rus A. (2023). From failure to meet the clinical endpoint to us Food and Drug Administration approval: 15th antisense oligonucleotide therapy approved Qalsody (Tofersen) for treatment of Sod1 mutated amyotrophic lateral sclerosis. Nucleic Acid Therapeutics 33, 234–237. doi: 10.1089/nat.2023.0027, PMID: 37581487

[ref176] Veitia R. A. Bottani S. Birchler J. A. (2008). Cellular reactions to gene dosage imbalance: genomic, transcriptomic and proteomic effects. Trends Genet. 24, 390–397. doi: 10.1016/j.tig.2008.05.005, PMID: 18585818

[ref177] Vicari S. Carlesimo G. A. (2006). Short-term memory deficits are not uniform in Down and Williams syndromes. Neuropsychol. Rev. 16, 87–94. doi: 10.1007/s11065-006-9008-4, PMID: 16967345

[ref178] Vickers T. A. Wyatt J. R. Burckin T. Bennett C. F. Freier S. M. (2001). Fully modified 2′ Moe oligonucleotides redirect polyadenylation. Nucleic Acids Res. 29, 1293–1299. doi: 10.1093/nar/29.6.1293, PMID: 11238995 PMC29745

[ref179] Vignoli A. Zambrelli E. Chiesa V. Savini M. La Briola F. Gardella E.. (2011). Epilepsy in adult patients with Down syndrome: a clinical-video Eeg study. Epileptic Disord. 13, 125–132. doi: 10.1684/epd.2011.0426, PMID: 21561839

[ref180] Wang S. Allen N. Vickers T. A. Revenko A. S. Sun H. Liang X.-H.. (2018). Cellular uptake mediated by epidermal growth factor receptor facilitates the intracellular activity of phosphorothioate-modified antisense oligonucleotides. Nucleic Acids Res. 46, 3579–3594. doi: 10.1093/nar/gky145, PMID: 29514240 PMC5909429

[ref181] Wegiel J. Gong C. X. Hwang Y. W. (2011). The role of Dyrk1A in neurodegenerative diseases. FEBS J. 278, 236–245. doi: 10.1111/j.1742-4658.2010.07955.x, PMID: 21156028 PMC3052627

[ref182] Wilton-Clark H. Yokota T., (2021). Casimersen for Duchenne muscular dystrophy, Drugs of Today Barcelona Spain: 1998, 57, 707–717. 34909800 10.1358/dot.2021.57.12.3352740

[ref183] Wurster C. D. Ludolph A. C. (2018). Antisense oligonucleotides in neurological disorders. Ther. Adv. Neurol. Disord. 11:1756286418776932. doi: 10.1177/1756286418776932 29854003 PMC5971383

[ref184] Yadav R. R. Sharma S. Joshi P. Wani A. Vishwakarma R. A. Kumar A.. (2015). Meridianin derivatives as potent Dyrk1A inhibitors and neuroprotective agents. Bioorg. Med. Chem. Lett. 25, 2948–2952. doi: 10.1016/j.bmcl.2015.05.034 26048785

[ref185] Yahya-Graison E. A. Aubert J. Dauphinot L. Rivals I. Prieur M. Golfier G.. (2007). Classification of human chromosome 21 gene-expression variations in Down syndrome: impact on disease phenotypes. Am. J. Hum. Genet. 81, 475–491. doi: 10.1086/520000, PMID: 17701894 PMC1950826

[ref186] Yamamoto T. Shimojima K. Nishizawa T. Matsuo M. Ito M. Imai K. (2011). Clinical manifestations of the deletion of Down syndrome critical region including Dyrk1A and Kcnj6. Am. J. Med. Genet. A 155, 113–119. doi: 10.1002/ajmg.a.33735 21204217

[ref187] Zamecnik P. C. Stephenson M. L. (1978). Inhibition of Rous sarcoma virus replication and cell transformation by a specific oligodeoxynucleotide. Proc. Natl. Acad. Sci. 75, 280–284. doi: 10.1073/pnas.75.1.280, PMID: 75545 PMC411230

[ref188] Zhang D. Li K. Erickson-Miller C. L. Weiss M. Wojchowski D. M. (2005). Dyrk gene structure and erythroid-restricted features of Dyrk3 gene expression. Genomics 85, 117–130. doi: 10.1016/j.ygeno.2004.08.021, PMID: 15607427

[ref189] Zhang L. Li D. Yu S. (2020). Pharmacological effects of harmine and its derivatives: a review. Arch. Pharm. Res. 43, 1259–1275. doi: 10.1007/s12272-020-01283-6, PMID: 33206346

